# The assembly dynamics of the cytolytic pore toxin ClyA

**DOI:** 10.1038/ncomms7198

**Published:** 2015-02-05

**Authors:** Stephan Benke, Daniel Roderer, Bengt Wunderlich, Daniel Nettels, Rudi Glockshuber, Benjamin Schuler

**Affiliations:** 1 University of Zurich, Department of Biochemistry, Winterthurerstrasse 190, 8057 Zurich, Switzerland; 2 ETH Zurich, Institute of Molecular Biology and Biophysics, Otto-Stern-Weg 5, 8093 Zurich, Switzerland

## Abstract

Pore-forming toxins are protein assemblies used by many organisms to disrupt the membranes of target cells. They are expressed as soluble monomers that assemble spontaneously into multimeric pores. However, owing to their complexity, the assembly processes have not been resolved in detail for any pore-forming toxin. To determine the assembly mechanism for the ring-shaped, homododecameric pore of the bacterial cytolytic toxin ClyA, we collected a diverse set of kinetic data using single-molecule spectroscopy and complementary techniques on timescales from milliseconds to hours, and from picomolar to micromolar ClyA concentrations. The entire range of experimental results can be explained quantitatively by a surprisingly simple mechanism. First, addition of the detergent *n*-dodecyl-β-D-maltopyranoside to the soluble monomers triggers the formation of assembly-competent toxin subunits, accompanied by the transient formation of a molten-globule-like intermediate. Then, all sterically compatible oligomers contribute to assembly, which greatly enhances the efficiency of pore formation compared with simple monomer addition.

## Abstract (web summary)

Pore-forming toxins are expressed as monomers and assemble into multimeric pores. Here, Benke *et al*. follow the kinetics of pore formation for the bacterial toxin ClyA with single-molecule methods and show that pore formation progresses through the assembly of oligomeric intermediates, rather than by the addition of monomers to a nascent pore.

## Graphical abstract

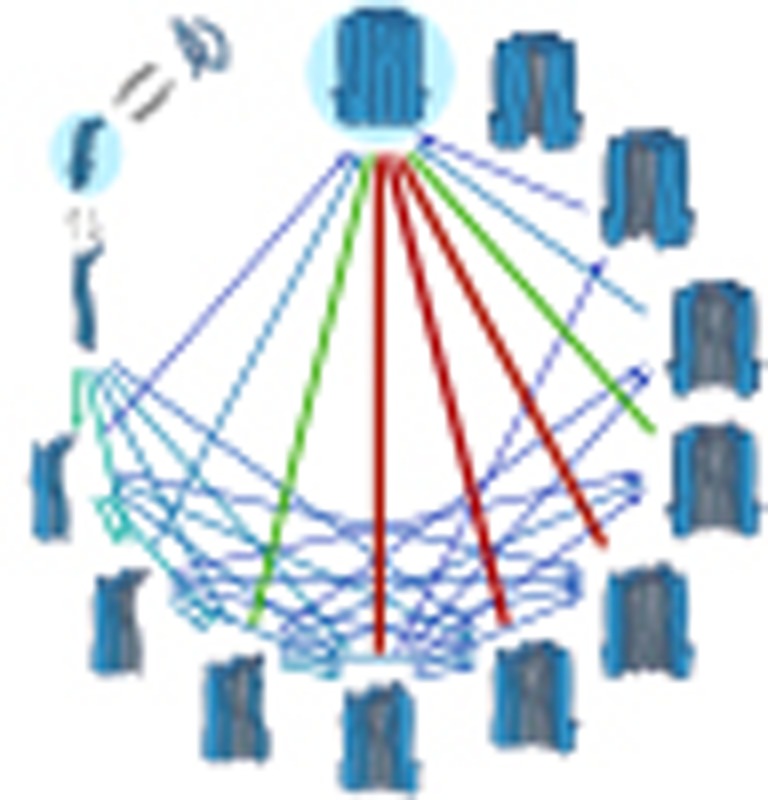

Pore-forming toxins (PFTs) are used both by prokaryotes and eukaryotes to kill cells by inserting channels into the target membranes^1^ and play an important role in bacterial virulence^2^. PFTs vary widely in pore size, structural complexity and delivery mechanisms^3^. Prominent examples are the homomeric 2.6-nm-wide α-hemolysin pore of *Staphylococcus aureus*^4^ and the 10-nm-wide complement membrane attack complex in humans built from five different proteins^5^. A common property of most PFTs is that they are produced as non-membrane-bound, soluble monomers that undergo a conformational rearrangement on binding to the target membrane, which triggers assembly into the oligomeric pore complex. From a structural point of view, there are two main classes of PFTs: α-PFTs and β-PFTs, which form membrane pores predominantly from α-helices or β-sheets, respectively^6^. The intriguing conformational changes that mediate pore assembly have been of great interest^7^,^8^,^9^,^10^,^11^,^12^,^13^, but the mechanisms of the underlying structural rearrangements and steps of pore assembly have remained challenging to elucidate, because the potentially very large number of kinetic intermediates are difficult to resolve experimentally.

Here we overcome this challenge by employing a combination of single-molecule fluorescence spectroscopy and complementary biophysical techniques to investigate the mechanism of pore formation of Cytolysin A (ClyA), a cytolytic toxin expressed in virulent *Escherichia coli* and *Salmonella enterica* strains^14^,^15^,^16^. ClyA from *E. coli* (Fig. 1a) is an α-PFT that forms ring-shaped homo-oligomeric pores with an inner diameter of 4 nm consisting of twelve 34-kDa subunits in the crystal structure^17^. It is currently the only α-PFT for which high-resolution structures of both the monomer^18^ and the pore complex^17^ are available. In addition, pore formation of ClyA does not require any receptor proteins and can also be triggered *in vitro* by detergents^7^,^19^, an ideal prerequisite for investigating the assembly mechanism of ClyA in detail.

ClyA monomers become assembly competent only after binding to membranes or detergent; it has been proposed that the monomer first undergoes a conformational change to a protomer (Fig. 1a), which then assembles to the dodecameric pore complex^7^,^17^. In its monomeric form, ClyA is an elongated, five-helix-bundle protein with a small β-turn on one end^18^. This β-tongue has been suggested to be the first part of the protein interacting with the membrane^18^ and to convert to α-helical structure on membrane binding^17^. The ensuing conformational changes involve more than half of all residues and include a reorganization of the hydrophobic core, transitions of β-sheets and loops to helices, and a large rearrangement of the long amino-terminal helix that forms the membrane-penetrating part in the assembled pore^17^ (Fig. 1a).

Clarifying the assembly mechanism of ClyA thus requires methods that are sensitive to conformational changes and allow the resulting structural heterogeneity of the ensemble to be resolved. Single-molecule Förster resonance energy transfer (FRET) in combination with microfluidic mixing^20^ allows intramolecular distance changes to be probed on timescales from milliseconds to hours over a wide range of protein concentrations. In combination with two-focus fluorescence correlation spectroscopy (2f-FCS)^21^, stopped-flow circular dichroism (CD) and photo-induced cross-linking^22^, our results enabled us to identify the kinetic mechanism for the initial conformational rearrangement in the monomeric protein and the following assembly of the cytolytic ClyA pore complex in the presence of detergent.

Our results show that the conformational transition of ClyA from the monomer to the assembly-competent protomer involves the formation of an off-pathway intermediate resembling a molten globule. Our data on the pore formation process support a kinetic mechanism according to which protomers first assemble into dimers and other oligomers; the association of all sterically compatible oligomers then dominates the assembly of higher oligomers and complete pores. The assembly steps leading to complete pores occur with a rate coefficient that is a hundred times greater than for the preceding association events, presumably due to the additional electrostatic steering effect provided by the second binding interface during closure of the ring-shaped pores. Compared with sequential monomer association, this mechanism greatly enhances the assembly efficiency and might also be applicable to other biomolecular assembly processes.

## Results

### Resolving conformational states by single-molecule FRET

**Figure 1:**
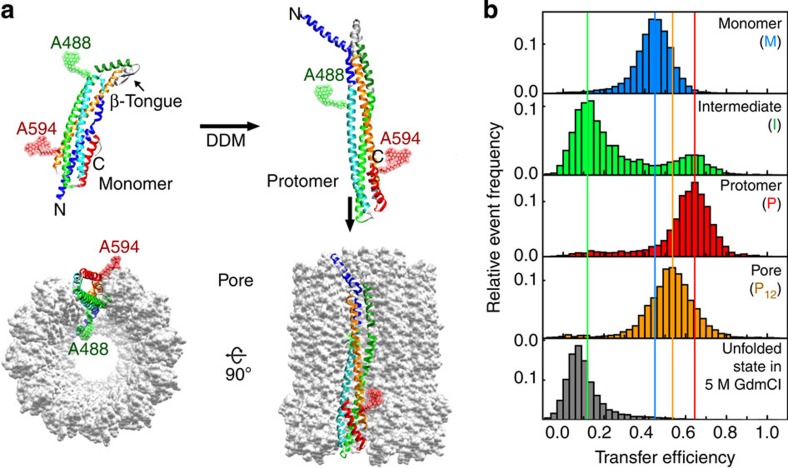
The different conformational states of labelled ClyA on mixing with DDM as observed by single-molecule FRET. (**a**) Crystal structures of the monomer and the ClyA protomer in the context of the pore complex (PDB code 1QOY^18^ and PDB code 2WCD^17^). The protomer conformation is represented by one pore subunit. N and C indicate the N- and carboxy termini of the protein, and A488 and A594 the positions labelled with Alexa Fluor 488 (position 56) and 594 (position 252), respectively. Structure representations were created with Chimera^63^ and Avogadro^64^, colour scheme according to Mueller *et al*.^17^ (**b**) Transfer efficiency histograms of the different ClyA species that can be distinguished by single-molecule FRET. The histograms (each containing ≳ 5,000 events) were normalized to an area of 1. The coloured vertical lines indicate the peak positions of the species in the transfer efficiency histograms. The histogram of the monomer (M) was recorded in the absence of DDM. The histograms of intermediate (I) and protomer (P) are from the kinetic measurements in the presence of DDM at a ClyA concentration of ~100 pM at 55 and 1,765 s, respectively, after mixing with DDM. For the histogram of the pore (P_12_), 5 μM ClyA with 1% labelled ClyA were incubated with DDM for 2 h and the formed pores were purified by size-exclusion chromatography according to Eifler *et al*.^7^ The resulting transfer efficiency is identical within uncertainty to what we observe in single-molecule kinetic measurements at times where oligomer formation is complete (Fig. 4a and Supplementary Fig. 8a). The difference between the transfer efficiencies of the oligomer state and the protomer can largely be assigned to acceptor dye quenching in the complex, rather than a difference in conformation (see Supplementary Table 1).

To study the conformational changes of ClyA during pore formation with FRET, we engineered a variant with additional cysteine residues for fluorophore labelling at positions 56 (helix B) and 252 (helix F) (Fig. 1a). These solvent-exposed positions were chosen based on the crystal structures of monomer^18^ and pore^17^, to achieve optimal separation of transfer efficiencies for the monomer, the protomer and pore conformation, and unfolded ClyA. ClyA was labelled site specifically with Alexa Fluor 488 at Cys56 as the donor and Alexa Fluor 594 at Cys252 as the acceptor dye (see Methods for details). The two wild-type cysteine residues at positions 87 and 285 were unreactive, as they are buried in the core of the folded monomer. The amino acid exchanges and labels have no significant influence on the haemolytic activity of ClyA (Supplementary Fig. 1), which attests to the full functionality of the labelled protein. Single-molecule experiments were performed with confocal detection of the FRET-labelled ClyA freely diffusing in solution at a concentration of ~100 pM (see Methods).

In keeping with the conditions used in previous studies on pore formation of ClyA^7^ and for crystallising the pore^17^, we triggered the conformational changes resulting in pore assembly with 0.1% (w/v) *n*-dodecyl-β-D-maltopyranoside (DDM). Figure 1 illustrates the different species populated by ClyA during pore formation and the corresponding transfer efficiency histograms. In the absence of detergent, single-molecule FRET measurements show a single peak with a mean transfer efficiency, ‹*E*›, of 0.46±0.03 (see Methods for details regarding uncertainties), as expected from the 5.5 nm *C*_α_—*C*_α_ distance of the labelling positions in the structure of the soluble monomer before conformational conversion^18^ (referred to here as the ‘monomer’). The addition of DDM triggers the depopulation of the monomer and the appearance of two additional peaks: one corresponding to a kinetic intermediate with ‹*E*›=0.12±0.03 and the other at ‹*E*›=0.67±0.03, which remains stable for hours at picomolar ClyA concentrations in the presence of DDM. Accordingly, we assign the latter peak to the protomer, the monomeric conformation competent for pore assembly. It is worth noting that the protomer had eluded direct observation in previous ensemble studies, because it rapidly assembles into pores at the micromolar ClyA concentrations used^7^, but it remains monomeric at the picomolar ClyA concentrations accessible in single-molecule experiments. The kinetic intermediate formed during protomer formation exhibits a similarly low transfer efficiency as ClyA denatured in 5 M guanidinium chloride (GdmCl) (Fig. 1b), suggesting substantial loss of tertiary structure in the intermediate (see ‘The nature of the intermediate’ below for details).

To initiate protomer association and pore formation without exceeding the low concentrations of labelled protein required for single-molecule detection, we increased the total ClyA concentration by adding a large excess of unlabelled wild-type protein ranging from 5 nM to 5 μM. (Statistically, the fraction of fluorescent pore complexes with more than one labelled subunit is between ~10^−1^ and 10^−4^ in this concentration span. We thus expect no detectable contribution of inter-subunit FRET to our measurements.) At these higher ClyA concentrations, we observe a fourth peak with ‹*E*›=0.53±0.03, which appears on the timescale reported for pore formation^7^ and agrees with the transfer efficiency obtained from ClyA pores purified by size-exclusion chromatography (Fig. 1b). The peak was thus assigned to labelled ClyA subunits within oligomeric assembly intermediates or intact pores (P_2–12_). In summary, by single-molecule FRET we can thus differentiate four conformational states involved in the assembly of ClyA: the monomer before its conformational conversion (M), a monomeric kinetic intermediate (I), the assembly-competent protomer (P) and the conformation of an individual ClyA subunit in the oligomeric assembly intermediates or in the pore (P_2–12_). By virtue of the very low subunit concentrations accessible in single-molecule experiments, we can additionally halt the reaction at the stage of the monomeric protomer. This separation of subpopulations enables us to dissect the role of these conformations in the assembly mechanism as a function of time.

### Kinetic mechanism of protomer formation

**Figure 2:**
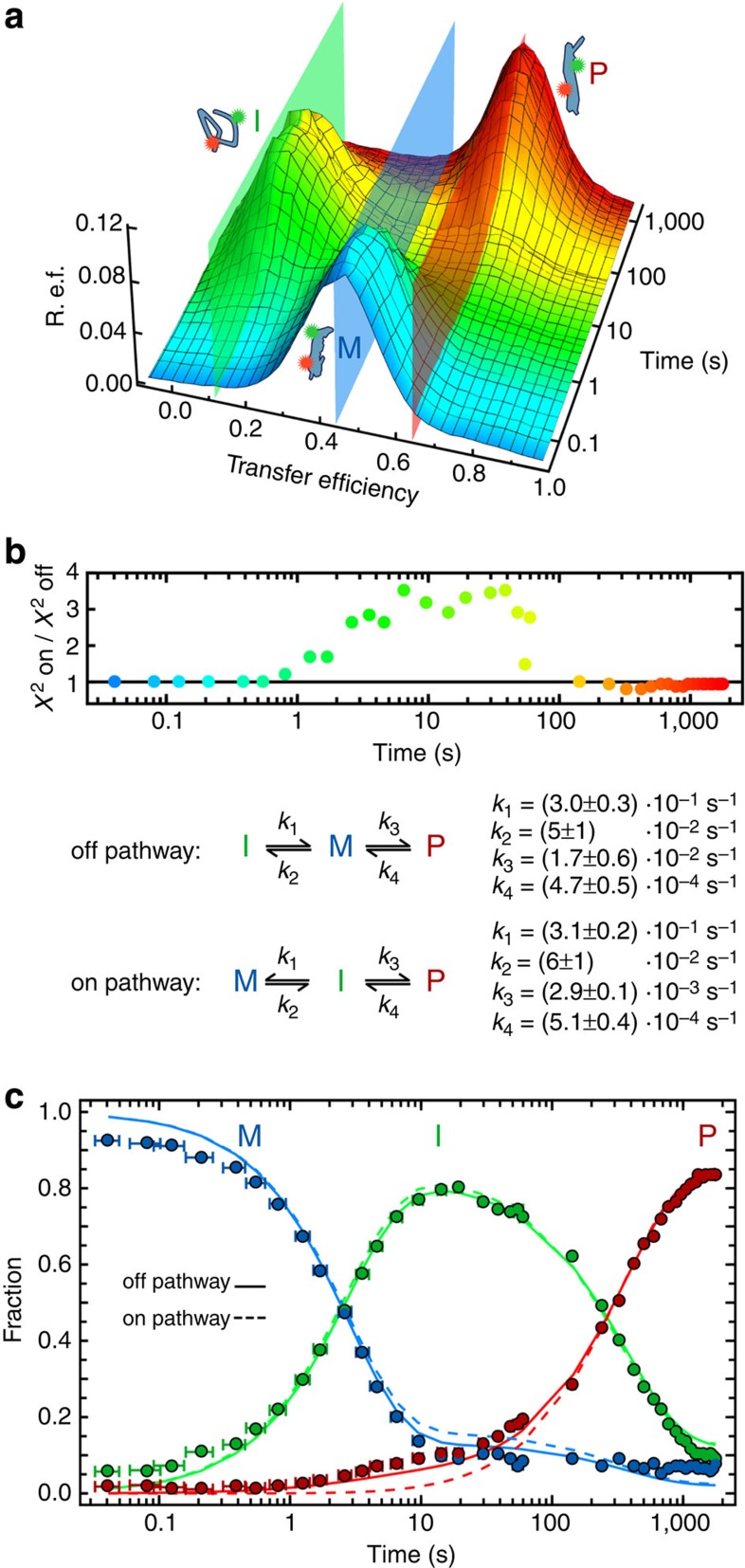
Kinetics of ClyA protomer formation. (**a**) Time series of transfer efficiency histograms measured for the monomer to protomer transition in 0.1% (w/v) DDM, combined from microfluidic and manual mixing experiments at short (<100 s) and long times (>40 s), respectively. Each histogram was normalized to an area of 1. The cartoons illustrate the monomer (M), intermediate (I) and protomer (P), and the coloured planes indicate the average transfer efficiencies of the peaks. R.e.f., relative event frequency. (**b**) Kinetic modelling of protomer formation. The histogram data were fit globally either with an off- or an on-pathway kinetic model (see Methods for details on the fitting procedure). Top: the *χ*^2^ ratio of the two fits for each histogram is shown (colour coded as in **a**), illustrating the better fit of the off-pathway model. Reconstructed histograms based on both the off-pathway and the on-pathway fit are shown in Supplementary Fig. 3. Bottom: schematic representation of the two models and the rate coefficients resulting from the fits to the data in **a**. For details on the uncertainties given, see Methods. (**c**) Population time courses of the different species according to three different fits of the histogram time series in **a**. Filled circles: histograms fit individually with free peak amplitudes. The error bars show the uncertainty in time in the microfluidic measurements due to Taylor dispersion^54^ (see Methods for details). Solid and dashed lines: populations from the fits according to the off- and the on-pathway models (see **b**), respectively. For details on error analysis of the global fits, see Supplementary Figs 2, 3 and 4.

We first focused on the monomer-to-protomer transition triggered by DDM at picomolar concentrations of ClyA, where protomer association and pore assembly are absent. As the formation of the low transfer efficiency intermediate is too rapid to be observed by manual mixing, we extended the accessible times by using a microfluidic mixing device with millisecond dead time^20^,^23^. With the combination of manual and microfluidic mixing, the entire reaction can be monitored from milliseconds to hours by recording transfer efficiency histograms that cover all stages of the kinetics (Fig. 2a). Already from the raw time series, several processes are evident: the decay of the monomer population within a few seconds, the transient appearance of the intermediate in the 10-s range and the emergence of protomer on the minutes timescale. As the transfer efficiency histograms resolve the subpopulations present at a given time, they allow detailed kinetic modelling of the populations of the individual species.

For a quantitative kinetic analysis, we make use of Gaussian and log-normal peak functions to empirically describe the peaks of the species in the transfer efficiency histograms based on the peak shapes observed under conditions where the respective population dominates (Fig. 1; see Methods and Supplementary Fig. 2 for details)^24^,^25^,^26^. The fractions of monomer, intermediate and protomer as a function of time resulting from fits with either free peak amplitudes (circles in Fig. 2c) or amplitudes constrained by kinetic models (lines in Fig. 2c) all show the pronounced transient population of the intermediate, with a maximum of ~80% at ~20 s (Fig. 2c). The subsequent decrease in the fraction of intermediate and the concomitant formation of the protomer might lead us to suspect that the intermediate is an obligatory step on the way to the protomer. However, it is well known that the two simplest mechanisms of a unimolecular reaction with a single intermediate (Fig. 2b) can be difficult to distinguish^27^,^28^,^29^. In the on-pathway mechanism, the monomer must first populate the intermediate to form the protomer; in the off-pathway mechanism, the intermediate and the protomer are both formed from the monomer but lack a direct connection (Fig. 2b). A hallmark of the on-pathway model is a lag in the formation of the protomer, but if the equilibration between monomer and intermediate is sufficiently rapid, the difference between the two kinetic mechanisms can be subtle, which turns out to be the case for the conversion of ClyA monomer to protomer.

Rather than introducing an intermediate data reduction step, we fit the two different kinetic models directly and globally to the entire time series of the 40 transfer efficiency histograms shown in Fig. 2a. By linking the respective peak areas to the populations predicted by the models (see Methods for details and Supplementary Fig. 2), this procedure allows the histogram time series to be reconstructed as a whole and then compared with the raw data (Supplementary Fig. 3). Comparison of the *χ*^2^-values of the two model fits to the individual histograms (Fig. 2b) shows that the fit quality of the two models only differs between 0.5 and 150 s, where protomer formation sets in earlier than expected for the on-pathway model (Fig. 2c). The better agreement with the off-pathway model is also robust in the presence of perturbations such as additional noise introduced into the data set (Supplementary Fig. 4). The question remains, however, what the conformational properties of the intermediate are.

### The nature of the intermediate

**Figure 3:**
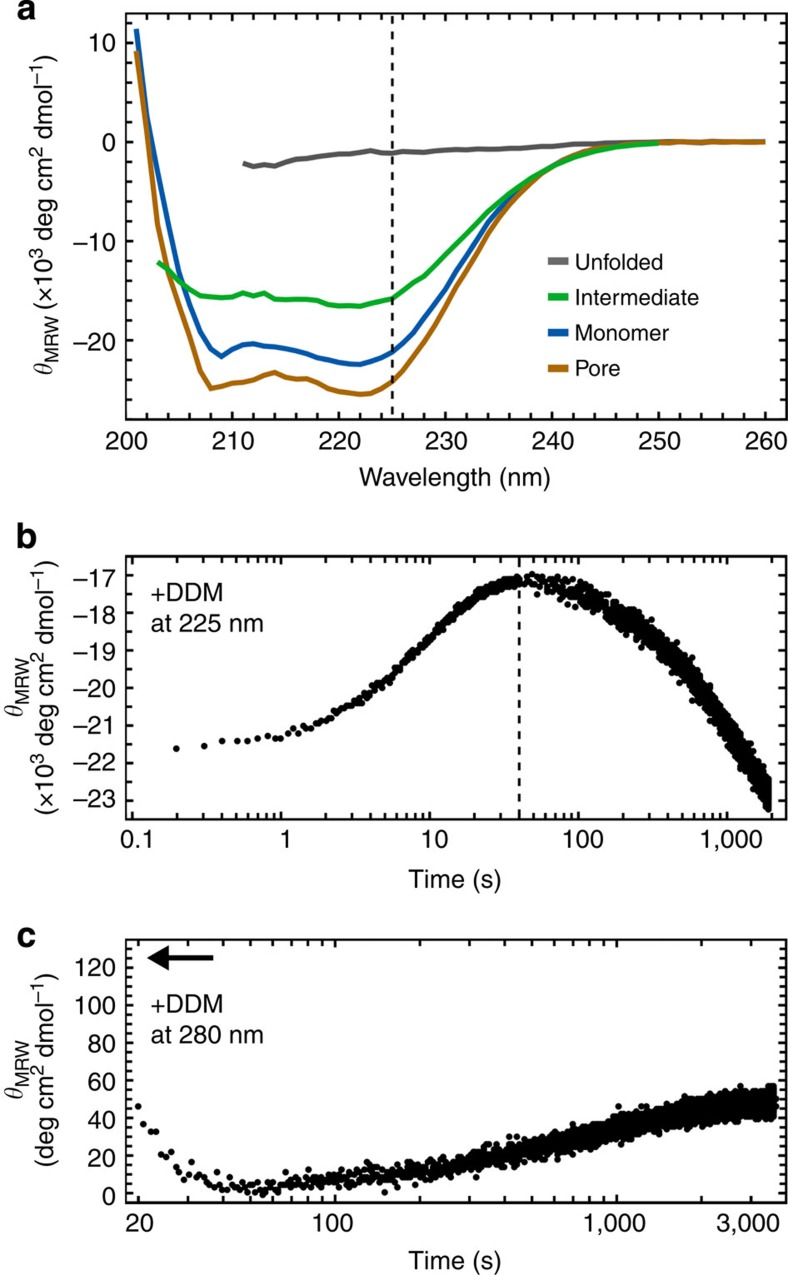
CD spectra of the different ClyA conformations and kinetic measurements. (**a**) Far-ultraviolet CD spectra of the conformational states of ClyAwt accessible with ensemble methods (that is, excluding the protomer). The spectrum of the intermediate is calculated from a reconstructed spectrum obtained by stopped-flow measurements 40 s after mixing with DDM and spectra of monomer and pore using the relative concentrations of the species at the point of maximum intermediate population determined by single-molecule FRET (Fig. 2c). The dashed line indicates the wavelength of the kinetic measurement in **b**. (**b**) Kinetic far-ultraviolet CD measurement at 225 nm of ClyAwt pore formation at 9 μM ClyA on mixing with DDM, combined from stopped-flow and manual mixing experiments. The manual mixing data were adjusted by +3 × 10^3^ deg  cm^2^ dmol^−1^ to match the level of the stopped-flow data. The dashed line indicates the time at which the spectrum of the intermediate was reconstructed in **a**. (**c**) Kinetic near-ultraviolet CD measurement at 280 nm of ClyAwt pore formation at 2.8 μM by manual mixing. The arrow indicates the level of the monomer signal to illustrate the signal drop during the dead time of the measurement.

The presence of a monomeric intermediate during ClyA pore assembly had already been evident in ensemble CD experiments based on the kinetics of changes in the ellipticity of ClyA at 225 nm: on mixing with DDM or erythrocyte membranes, a state with less negative CD signal compared with the monomer (that is, with less secondary structure) was populated before the formation of the protomer^7^,^30^. Here we used multi-wavelength stopped-flow and manual-mixing CD experiments to probe the secondary structure content and tertiary packing of the intermediate of unlabelled wild-type protein (ClyAwt) in DDM (Fig. 3). The resulting time of maximum population of the intermediate of ~40 s is within about a factor of two of the single-molecule measurements, indicating that the additional cysteine residues and the labels affect the kinetics only marginally (Supplementary Fig. 5 and Fig. 2b). The reconstructed far-ultraviolet CD spectrum of the intermediate indicates that it retains about 75% of the ellipticity at 225 nm compared with the folded monomer (Fig. 3a,b). For comparison, ClyA denatured in 5 M GdmCl shows <5% of the CD signal (Fig. 3a). The CD spectrum of the assembled pore exhibits a more pronounced α-helical signal than the folded monomer (Fig. 3a), as expected from the conversion of the β-tongue region into α-helical segments^17^,^18^. Kinetic near-ultraviolet CD measurements at 280 nm show a transient signal drop to almost zero ellipticity at ~40 s (Fig. 3c), indicating the loss of tertiary structure in the intermediate.

From the single-molecule FRET measurements, we obtained complementary information on the dimensions of the intermediate. Based on its transfer efficiency of ‹*E*›=0.12±0.03 (slightly higher than that of ClyA denatured in 5 M GdmCl, ‹*E*›=0.07±0.03, Fig. 1b), we estimate a radius of gyration of 6 nm using a simple polymer model for the intramolecular distance distribution^26^. A ClyA variant with cysteine residues 87 and 285 replaced by alanine (ClyAΔCys) populates the intermediate to ~50% at equilibrium in 0.1% DDM at picomolar ClyA concentrations, which additionally enables us to estimate the Stokes radius, *R*_S_, of the intermediate with subpopulation-specific fluorescence correlation spectroscopy to be 4.9±0.1 nm (Supplementary Fig. 6 and see Methods for details and calculation of uncertainties), only slightly less than for ClyA unfolded in 5 M GdmCl (5.3±0.1 nm). Increasing amounts of DDM stabilize the intermediate relative to the protomer and lead to a slight additional expansion (Supplementary Fig. 7). Taken together, the CD experiments indicate the presence of substantial secondary structure but lack of defined tertiary structure in the intermediate, while single-molecule FRET and correlation spectroscopy point towards a denatured structure. The intermediate thus resembles what is commonly referred to as a molten globule state^31^,^32^.

### Single-molecule FRET measurements of ClyA oligomerization

**Figure 4:**
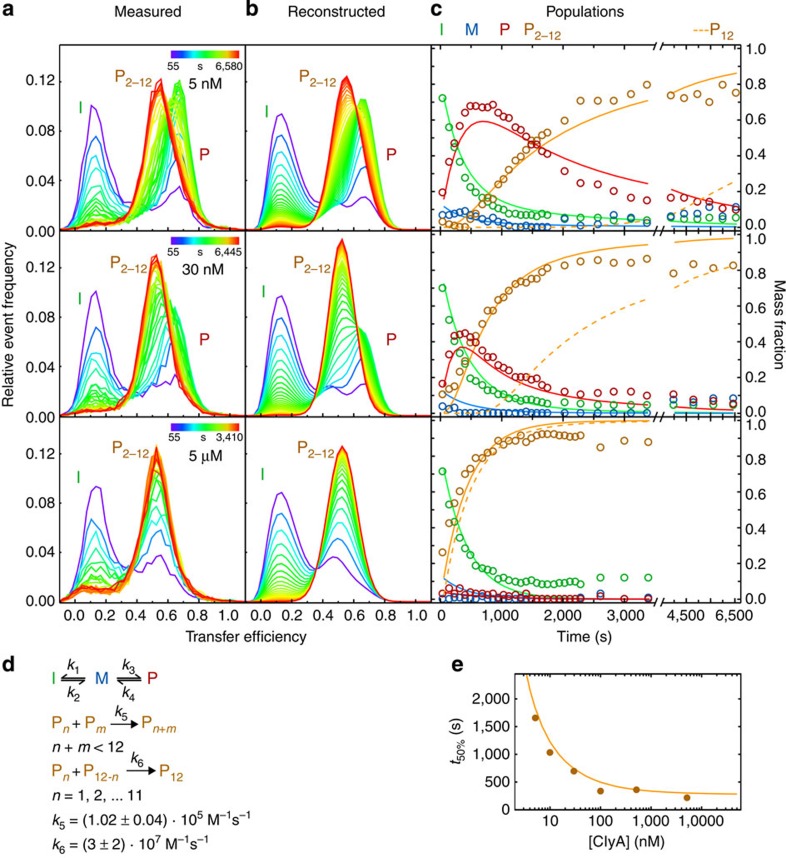
Kinetics of ClyA pore formation at different total ClyA concentrations followed by single-molecule FRET. (**a**) Measured transfer efficiency histogram time series after manual mixing. Each coloured line represents one histogram (area normalized to 1) at a certain time after starting the reaction (colour code shown in the upper right of each panel). Most of the monomer depopulates during the dead time of the experiment, and thus no pronounced monomer peak is observed. (**b**) Reconstructed histograms according to the pore formation model shown in **d**. (**c**) Population time courses of the different species according to two different types of analysis of the histogram time series. Circles: populations from individual fits of the histograms with peak amplitudes as free parameters, and peak positions and widths as shared (global) fit parameters. Solid lines: populations from a global fit of all 217 histograms from all ClyA concentrations according to the non-sequential assembly model with two rate coefficients (see **d**). As the different oligomers cannot be discriminated in the histograms, the population P_2–12_ represents the total population of all protomers in oligomers. The dashed line shows the population of complete pores as predicted by the model (see Supplementary Fig. 9c for the other oligomers). See Methods section for details on the fitting procedure. Data for ClyA at 0.1, 10, 100 and 500 nM were also included in the global fit (Supplementary Fig. 8). I, intermediate; P_2–12_, oligomeric species; P, protomer; M, monomer. (**d**) Schematic of the non-sequential assembly model of pore formation. Protomer formation occurs according to an off-pathway model (Fig. 2); oligomerization of protomers and assembly with other oligomers all occur with the same rate coefficient (*k*_5_) if they lead to incomplete pores; formation of complete pores occurs with a different rate coefficient (*k*_6_). See Methods for details on error calculation. (**e**) Dependence of pore formation kinetics on ClyA concentration. Plotted is *t*_50%_ (time when 50% of the ClyA molecules are in an oligomeric state), versus the total concentration of ClyA subunits, according to the populations from the free fit (filled circles) and as predicted by the pore assembly model (**d**; solid line).

Next, we focused on the assembly of the ClyA pore complex. A key aspect for identifying the kinetic mechanisms of higher-order reactions such as multimolecular assembly is their dependence on protein concentration^33^. Pores are not yet formed in DDM at ClyA concentrations of 100 pM (Supplementary Fig. 8c), and pore formation kinetics were previously found to be concentration independent in the low micromolar range^7^, indicating that collisional encounter is no longer rate limiting. We thus studied the process between these extremes, at ClyA concentrations from 5 nM to 5 μM, by varying the amount of unlabelled ClyAwt added to samples of 100 pM FRET-labelled protein. We observed a pronounced concentration dependence of the kinetics (Fig. 4a–c,e and Supplementary Fig. 8a). At the reaction times where pore formation is observed, the monomer (M) is already depopulated virtually completely, but the decay of the intermediate population (I) and the conversion of the protomer (P) to oligomers (P_2–12_) is clearly visible in the transfer efficiency histograms. The rate of decrease in the population of the intermediate is independent of ClyA concentration (Fig. 4c and Supplementary Fig. 8a), a strong indication that only protomers are assembly competent. However, with increasing ClyA concentration, less of the transient formation of protomer is observed and the rate at which oligomers accumulate increases (Fig. 4c and Supplementary Fig. 8a). At 5 μM ClyA, the oligomerization kinetics approach the concentration-independent regime where protomer formation becomes rate limiting^7^ (Fig. 4e).

Although the single-molecule FRET measurements allow us to distinguish monomer, intermediate, protomer and oligomer, they cannot discriminate the different oligomeric species on the way to the pore, indicating that during assembly neither the conformation of the subunits nor the local environment changes significantly for the structural elements probed by single-molecule FRET. To identify the mechanism of oligomerization and pore formation, we thus obtained additional information on the oligomeric species from photo-induced cross-linking^22^ and kinetic 2f-FCS^21^ experiments.

### Oligomeric species populated during pore formation

**Figure 5:**
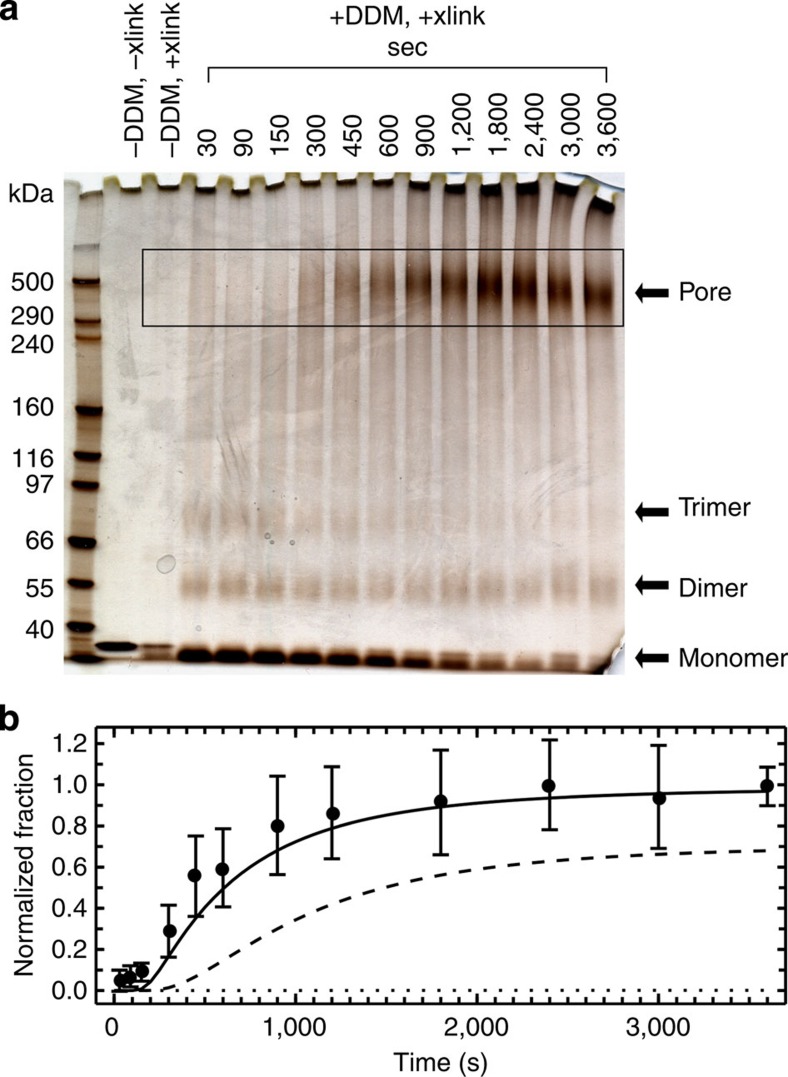
Kinetics of ClyA pore assembly at 500 nM ClyAwt followed by photo-induced cross-linking. (**a**) Samples were cross-linked by photo-induced cross-linking of unmodified proteins (PICUP)^22^,^52^ (see Methods) after incubation with DDM for different times and analysed by SDS–PAGE. The image shows the silver-stained polyacrylamide gel. Lane 1: molecular weight marker (molecular masses indicated on the left), lane 2: monomeric ClyA (no addition of DDM or crosslinker), lane 3: cross-linked monomeric ClyA in the absence of DDM (only cross-linker added), lane 4 to 15: cross-linked ClyA samples after incubation with DDM for the time intervals indicated on top of the respective lane. The rectangle indicates the region used for the analysis in **b**. The monomer band becomes a double band after cross-linking, which we attribute to internal cross-linking that leads to a more compact unfolded state. Owing to incomplete cross-linking, residual populations of small oligomers are still detected after denaturation in SDS. (**b**) Time course of pore formation based on the fluorescence of Coomassie-stained gel bands (see Methods). The means and s.d. of the band intensities of three experiments (filled circles and error bars) were normalized to the mean of the cross-linking control as the minimum and the mean of the highest intensity as the maximum. Note that the fraction of pores (see **b**) is normalized to the pore band intensity at 3,600 s, where pore formation is complete under these conditions (Fig. 6). The lines represent the time courses of the formation of complete pores predicted by three different models: the non-sequential addition model with two rate coefficients (solid line), the non-sequential addition model with a single rate coefficient (dashed line) and the linear addition model with a single rate coefficient (dotted line), using the kinetic parameters obtained from the single-molecule FRET data (Figs 2 and 4).

Previous cross-linking experiments using an amine-reactive reagent showed a decrease in monomer population in the expected time range but did not resolve oligomeric species^7^. Here we thus used cross-linking by light-induced radicals, which allows cross-linking reactions to be performed within seconds and is therefore ideally suited for following the kinetics of transiently populated oligomers in protein assembly without inducing artificial association^22^,^34^. ClyAwt (500 nM) was cross-linked at different times after triggering the assembly reaction with DDM by exposure to laser light at 490 nm in the presence of tris-bipyridylruthenium(II) and ammonium persulfate; the cross-linking products were analysed by SDS–polyacrylamide gel electrophoresis (PAGE) (Fig. 5a). In addition to the monomer band at ~35 kDa, intermolecular cross-linking leads to three discernible bands at apparent molecular masses of about 50, 80 and 400 kDa. The band at about 400 kDa first appears after ~300 s and is in the range expected for the complete dodecameric pore (414 kDa). The intensity of the band at 80 kDa, which is presumably a trimer compacted by cross-linking, starts to decrease at the same time as the pore band appears. The band at 50 kDa, most likely to be a compacted dimer, decreases only slightly over time. A quantitative interpretation of the dimer and trimer bands is problematic, because they could also result from incomplete cross-linking of larger assemblies. Oligomers larger than the trimer were not detectable, indicating that these species are hardly populated during assembly. Quantitative densitometric analysis of the appearance of the band at 400 kDa shows that significant pore formation occurs already within 300 s (Fig. 5b), an important constraint for identifying the appropriate assembly mechanism.

**Figure 6:**
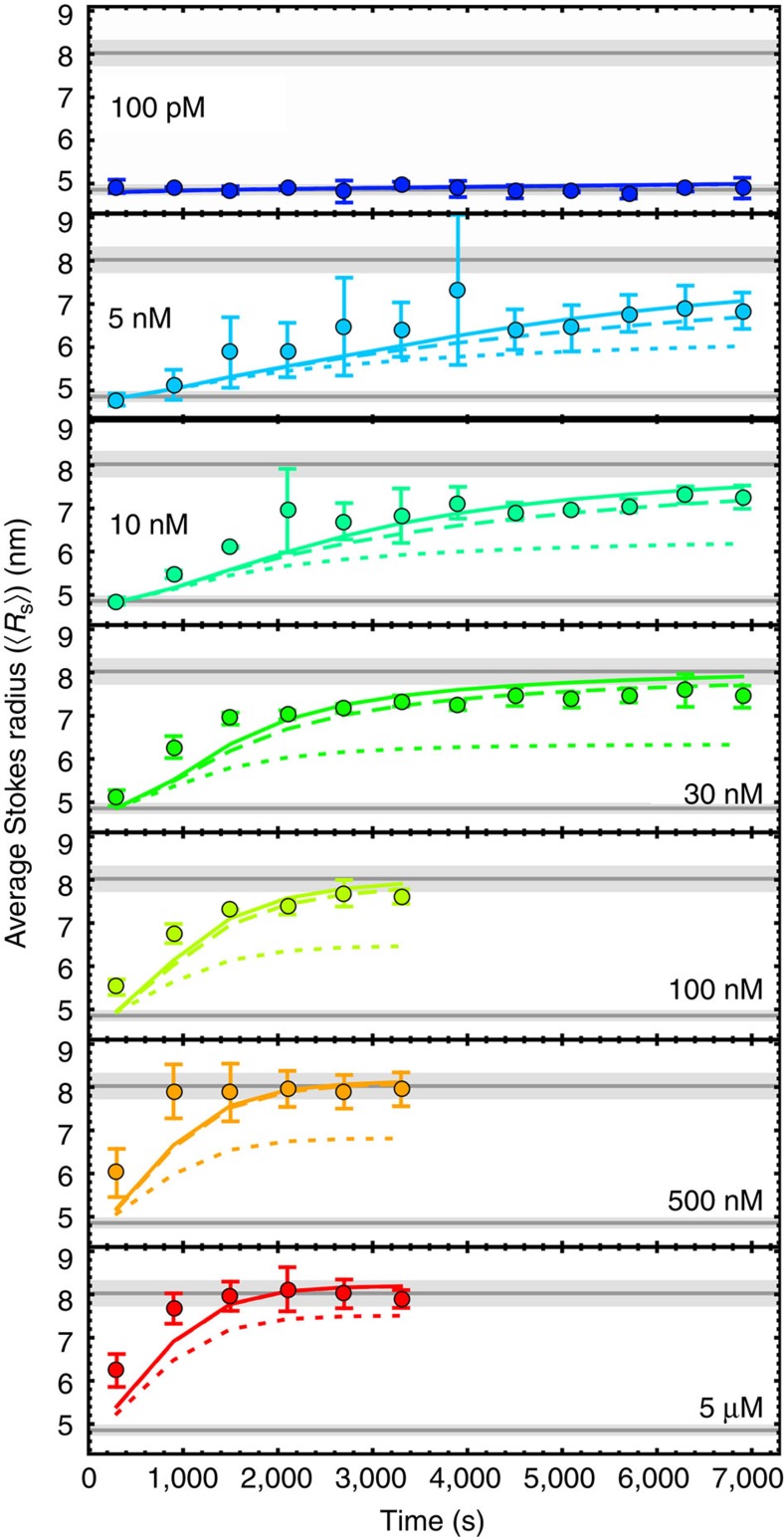
Changes in hydrodynamic radius during pore formation. The average Stokes radius, ‹*R*_S_›, as a function of time during pore formation reactions at different ClyA concentrations was determined by 2f-FCS^21^ (see Methods). Filled circles and error bars represent mean and s.d. of three measurements. The lines in each graph represent the time courses of ‹*R*_S_› predicted by three different models: the non-sequential assembly model with two rate coefficients (solid lines, see Fig. 4), the non-sequential assembly model with a single rate coefficient (dashed lines) and the linear protomer addition model with a single rate coefficient (dotted lines) (see Results and Methods for details), using the kinetic parameters obtained from the fit to the single-molecule FRET data (Figs 2 and 4). The grey lines and shaded bands represent the means and s.d. of the Stokes radii for the protomer (0.1 nM ClyA, *R*_S_=4.9±0.1 nm) and the pore complex (5 μM ClyA, *R*_S_=8.0±0.3 nm) from three independent measurements each.

With 2f-FCS^21^, we can probe the dimensions of ClyA oligomers also at lower concentrations than feasible for cross-linking, and independently of cross-linking efficiency, analogous to previous applications of FCS for monitoring peptide or protein assembly^35^,^36^. Although 2f-FCS cannot resolve individual oligomers, the kinetics of the change in the Stokes radius averaged over all species present in the sample, ‹*R*_S_›, can be extracted for a wide range of ClyA concentrations (Fig. 6). After the start of the assembly reaction, ‹*R*_S_› increased from the protomer value of 4.9±0.1 nm towards the 8.0±0.3 nm of the complete pore for all ClyA concentrations where oligomerization was observed (Fig. 4 and Supplementary Fig. 8). Pore formation accelerated with increasing concentration up to ~100 nM ClyA. Below this concentration, ‹*R*_S_› remained lower than 8.0 nm during the observation time, indicating that not all ClyA molecules had been incorporated into complete pores within 2 h. Furthermore, the increase in ‹*R*_S_› is much slower than the formation of the sum of all oligomeric species (P_2–12_) as observed in the corresponding FRET measurements (Fig. 4a and Supplementary Fig. 8). This finding indicates that the P_2–12_ population at these ClyA concentrations not only includes complete pores but also a significant fraction of smaller oligomers.

### Kinetic mechanism of ClyA pore formation

By combining our results on pore formation from single-molecule FRET, 2f-FCS and cross-linking experiments, we arrive at the following constraints for the assembly mechanism of ClyA in DDM. First, oligomerization starts only from the protomer (single-molecule FRET, Fig. 4). Second, the model must lead to complete pores as the dominant product for concentrations above 100 nM within ~1 h (2f-FCS and cross-linking, Figs 5 and 6), and at 500 nM ClyA, a significant pore population must be present already after 300 s (cross-linking, Fig. 5). Third, the model must be able to explain the pronounced concentration dependence of the rate of pore formation observed by 2f-FCS (Fig. 6). These stringent constraints and the diverse set of data that need to be described simultaneously thus permit a comprehensive test of alternative assembly mechanisms.

**Figure 7:**
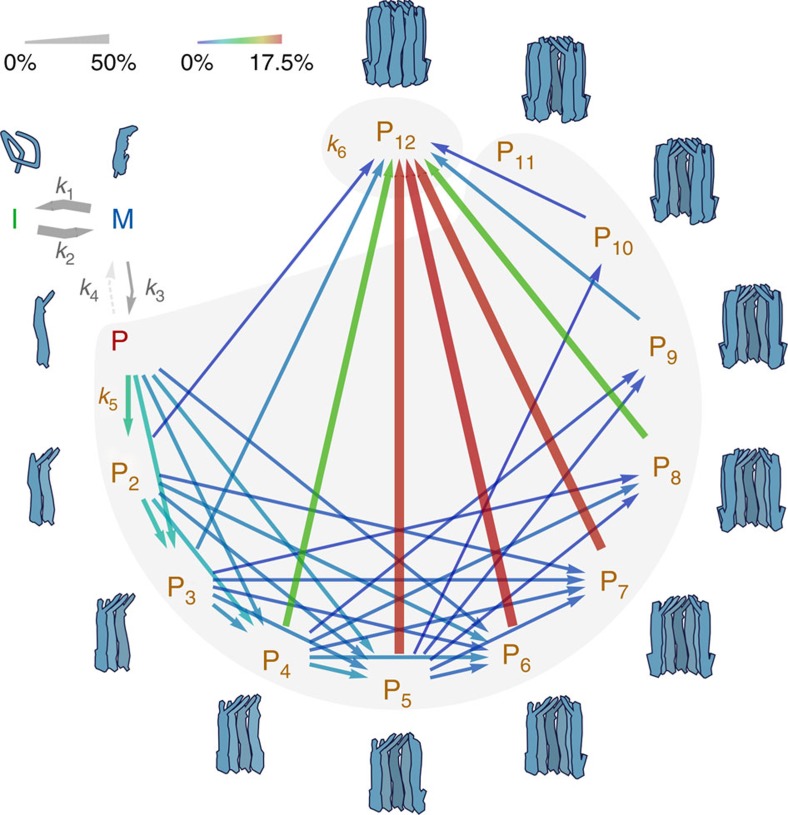
Pathways of ClyA pore formation. Shown is the mass flux (arrows) through the states of the non-sequential assembly model with two rate coefficients (Fig. 4d) for a ClyA concentration of 5 μM according to the fit shown in Fig. 4 and Supplementary Fig. 8. Each arrow represents either a conformational change (grey arrows between I, M and P) or an oligomerization step (P to P_12_). For example, the arrow between P_5_ and P_12_ represents the association of pentamers (P_5_) and heptamers (P_7_) to form complete pores (P_12_). Widths and colours of the arrows correspond to the normalized flux from one state to another according to the scales shown at the top left. As the pre-equilibrium between monomer and intermediate accounts for the majority of the total flux, the flux was normalized independently for the pre-equilibrium (including M, I and P) and for the pore assembly process (including P to P_12_). For clarity, only paths contributing more than 1% to the total flux are shown (except for the P to M transition). The relative fluxes in this model are remarkably insensitive to the ClyA concentration. Between the lowest (5 nM) and the highest (5 μM) ClyA concentration, the maximum difference in flux for a single path in pore assembly is 2% of the total flux. I, intermediate; M, monomer; P, protomer; P_i_, oligomeric species.

Arguably, the simplest conceivable model for ClyA assembly would resemble a sequential isodesmic polymerization reaction that involves only the addition of individual protomers, with identical rate coefficients for all steps, until the dodecamer is reached. Even though such a mechanism is sufficient for describing the time course of the transfer efficiency histograms, which report on the fraction of ClyA in oligomers of all sizes, it predicts pore populations (Supplementary Fig. 9a) that are substantially below the population of complete pores detected by cross-linking (Fig. 5b) and the change in ‹*R*_S_› observed with 2f-FCS (Fig. 6). However, a simple modification of the model leads to better agreement with all measurements: as the protomer and all oligomers with 2 to 11 subunits contain equivalent association interfaces, there is no reason to assume that the pores grow only by the addition of individual protomers; instead, growth is more likely to proceed also by the association of all sterically compatible oligomers^37^. Assuming again the same rate coefficient for all oligomerization steps still leaves a significant discrepancy between the predicted (Supplementary Fig. 9b) and experimentally observed fraction of pores formed when the reaction approaches saturation (Figs 5b and 6). However, this disagreement can be resolved by introducing a second, higher rate coefficient for the final assembly step, the association of any two complementary oligomers that lead to a complete dodecameric pore (Figs 4d and 7).

We used this non-sequential assembly model (Figs 4d and 7) to globally fit the entire set of single-molecule FRET efficiency histogram time series monitored from 55 s to 2 h, at all ClyA concentrations from 100 pM to 5 μM, by numerically solving the corresponding system of differential equations (see Methods for details). The rate coefficients of intermediate and protomer formation are well-defined by the values obtained independently in the absence of oligomerization (Fig. 2), thus leaving the two rate coefficients for pore assembly as the only remaining adjustable parameters. The global fits with the model are displayed both as reconstructed time series of transfer efficiency histograms (Fig. 4b), and in terms of the resulting kinetics of all populations in comparison with the values obtained from fitting the histograms individually (Fig. 4c and Supplementary Fig. 8a; see Methods for details). The non-sequential kinetic mechanism describes the ClyA concentration dependence of the assembly kinetics surprisingly well and successfully predicts the formation of a significant fraction of complete pores for all ClyA concentrations within the 2-h duration of the experiments (except at 100 pM, where pores do not form during the observation time; Supplementary Fig. 8c). The model also predicts correctly that the transiently populated fractions of protomer and oligomeric intermediates decrease with increasing ClyA concentration and become virtually undetectable at 5 μM (Fig. 4a–c). A rate coefficient for pore closure, *k*_6_, of about ≥10^7^ M^−1^ s^−1^ is required for the predicted kinetics of the formation of complete pores to match the cross-linking data (Fig. 5b), two orders of magnitude faster than for the preceding steps with *k*_5_=(1.02±0.04) × 10^5^ M^−1^ s^−1^. Finally, the predictions of this model also agree well with the increase in ‹*R*_S_› during pore formation determined with 2f-FCS (Fig. 6).

In summary, the kinetic mechanism presented here (Fig. 7) is able to describe the single-molecule FRET experiments for protomer formation (Fig. 2) and assembly (Fig. 4a and Supplementary Fig. 8a), the cross-linking data (Fig. 5b) and the 2f-FCS results (Fig. 6), including their concentration dependencies, with a single set of kinetic parameters. A remarkable aspect of the mechanism is that even though the populations of any one oligomeric species are small (Supplementary Fig. 9c), their total contribution to the kinetics is substantial and accelerates the formation of complete pores compared with mechanisms that assume only protomer addition, as suggested previously for virus capsid assembly^37^. The corresponding reactive fluxes are illustrated in Fig. 7. We would like to stress that in spite of the large number of microscopic pathways entailed by the combinatorics of oligomer association, the rate coefficients for all assembly steps are identical (*k*_5_), with the exception of the faster formation of the final dodecameric pore (*k*_6_). Note that the oligomerization steps were assumed to be irreversible, as no significant disassembly of already formed oligomers could be observed in dilution experiments on the timescale investigated (Supplementary Fig. 11). This observation may not be surprising given the large number of hydrogen bonds and salt bridges at the subunit interface^17^. For the analysis presented here, we limited the number of subunits in the complete pore to 12, as in the crystal structure, but extending the model to slightly smaller or larger complexes, as suggested by some experiments^7^,^19^,^38^, is straightforward and leads to very similar results.

## Discussion

The formation of cytolytic pores by ClyA starts with a large conformational rearrangement of the monomeric protein, followed by the assembly to a complex of about 12 subunits^7^,^17^,^19^,^38^. Based on a large data set obtained with biophysical techniques that probe complementary aspects of the reaction, in particular by virtue of the resolution of conformational subpopulations by single-molecule FRET and the sensitivity of 2f-FCS, we were able to resolve the dynamics with sufficient detail to propose a coherent kinetic mechanism of the entire assembly process in DDM, including both the initial conformational changes at the level of the monomeric subunits and their subsequent oligomerization.

The monomeric intermediate accumulating during the conformational change of ClyA exhibits the properties commonly assigned to molten globules^31^,^32^. Interestingly, an intermediate with reduced tertiary structure compared with the monomer was also observed in experiments with erythrocyte ghost membranes^7^, suggesting that it is not merely a side reaction caused by the presence of DDM, but that it is also relevant for ClyA assembly in membranes^9^. Conformational states showing the characteristics of a molten globule have been proposed to be important in the pore formation of several other PFTs (colicin A^39^, diphtheria toxin^40^,^41^ and equinatoxin II^42^), and it might seem intuitive that a denatured intermediate facilitates the transition between two rather differently folded states. However, our finding that the formation of this intermediate is not on the pathway to the protomer rather supports the model proposed based on the crystal structures that the large conformational changes during protomer formation occur without global unfolding of the monomeric subunit^17^.

Our results indicate that once the protomer is formed, pore assembly can be explained via an intriguingly simple mechanism (Fig. 7). Protomers dimerize, associate with other oligomers and existing oligomers join, all without detectable conformational adjustments in the four-helix bundle of the subunit. As illustrated in Fig. 7, the formation of pores by sequential addition of single protomers is negligible. Higher oligomers are mainly formed by the dimerization of smaller oligomers and the majority of pores assemble from species ranging from tetramers to octamers. This effect is reflected in the bimolecular rate coefficient for pore closure in the final assembly step (*k*_6_) being greater than the one for the formation of smaller oligomers (*k*_5_). Note that a combination of sequential monomer addition with a final, fast monomolecular ring closure step is insufficient to account for this increase in reactive flux. Of course, more complex models with a larger number of distinct rate coefficients can currently not be excluded, but in view of the architecture of the ClyA pore with completely equivalent binding interfaces between the subunits and variations in translational diffusion coefficients by less than a factor of two from protomer to pore (Supplementary Fig. 10), the approximations used in the present model appear reasonable.

What could be the molecular origin of the higher rate coefficient for pore closure (*k*_6_)? The rate coefficient *k*_5_=(1.02±0.04) × 10^5^ M^−1^ s^−1^ is in the range typical of macromolecular association reactions in free solution that are not limited by large conformational changes^43^. An important contribution to protein–protein association rates comes from long-range electrostatic interactions. The highly favourable electrostatics between the large number of complementary charges at the interfaces between two ClyA subunits^17^ are thus expected to contribute substantially to the observed rates. In the step of pore closure from two compatible oligomers, not only two but four interfaces are involved, and the electrostatic interactions that favour association are expected to be amplified accordingly, leading to an increased rate coefficient^44^. The combination of this electrostatic steering effect with the non-sequential assembly of all compatible oligomers (Fig. 7) may be a critical mechanism for enhancing the efficiency of pore assembly in the context of the limited reservoir of toxin subunits in the natural environment.

Prompted by previous biochemical^7^,^30^ and structural^17^ investigations, we used the detergent DDM as a membrane mimic to trigger ClyA pore formation. What are the possible differences of the mechanism compared with assembly in lipid bilayers? Eifler *et al*.^7^ observed that the formation of the protomer is about tenfold faster in erythrocyte membranes than in DDM. This difference could be influenced by the additional kinetic step of association of the subunits with the membrane; by different relative conformational stabilities of monomer, intermediate and protomer in the lipid bilayer; the restriction to two-dimensional diffusion in the membrane; or the additional pre-alignment of the pore subunits. However, as the subunit interfaces that drive association in the membrane are the same as in free solution, it seems probable that the fundamental mechanism of assembly, in particular the role of the association of smaller oligomers identified here, will be similar in both cases. The presence of a molten globule-like intermediate under both conditions also suggests similar mechanisms. Nevertheless, extending the investigations of ClyA to assembly in intact membranes is an essential next step. Recently, Vaidyanathan *et al*.^9^ monitored ClyA pore formation by haemoglobin release from erythrocytes lysed by the toxin. They tested two kinetic models of assembly with their data, a linear protomer addition and a non-sequential addition model, both with the same rate coefficients for all steps, and reported slightly better agreement with the former. It will be of great interest to combine such experiments with the biophysical methods employed here to obtain a complete picture. A remaining challenge, however, is the identification of the physiologically most relevant membrane system and lipid composition; as ClyA is exported into the periplasm after synthesis, assembly can already take place in the bacterial outer membrane and not only on the target cell^45^.

The mechanism of ClyA assembly differs from the previous proposals for β-PFTs; for example, the sequential monomer addition suggested for α-haemolysin^13^, the dimer addition for γ-haemolysin^11^ and the dimer addition with a last monomer addition step for anthrax toxin^12^. However, the demands on the number and quality of experimental constraints required to distinguish assembly mechanisms can be considerable, making an unequivocal distinction between kinetic models difficult. The approach of integrating multiple single-molecule and ensemble techniques over a broad range of conditions for a global kinetic analysis presented here may be useful not only for the mechanisms of pore formation, but for a wide range of biomolecular association reactions, including large hetero-oligomeric complexes and virus assembly^37^,^46^.

## Methods

### Protein preparation

ClyA variants (with amino acid exchanges Q56C/E252C and Q56C/C87A/E252C/C285A) were produced by site-directed mutagenesis of the pClyA plasmid (on the basis of the pET11a vector (Merck Millipore)) that codes for N-terminally His_6_-tagged ClyA^7^, using the QuikChange protocol (Stratagene) with the following primers (synthesized by Microsynth): Q56C-forward: 5′- CTTTAAACAGGAGTATTCATGCGCAGCCTCCGTTTTAGTCG -3′; Q56C-reverse: 5′- CGACTAAAACGGAGGCTGCGCATGAATACTCCTGTTTAAAG -3′; E252C-forward: 5′- CCGAAATAGCCGCCATCGGTTGCATAAAAACTGAAACTGAAAC -3′; E252C-reverse: 5′- GTTTCAGTTTCAGTTTTTATGCAACCGATGGCGGCTATTTCGG -3′; C87A-forward: 5′- CAAACAGTGTATGAATGGGCTGGTGTTGCGACGC -3′; C87A-reverse: 5′- GCGTCGCAACACCAGCCCATTCATACACTGTTTG -3′; C285A-forward: 5′- GCCAAAAAAATGATTAACACCGCTAATGAGTATCAGAAAAG -3′; C285A-reverse: 5′- CTTTTCTGATACTCATTAGCGGTGTTAATCATTTTTTTGGC -3′.

The amino acid numbering follows that of the wild-type protein, ClyAwt. Protein production was conducted in *E. coli* Tuner DE3 (Merck Millipore) in LB medium supplemented with 150 mg l^−1^ ampicillin at 20 °C for 12 h. Proteins were purified by nickel chelate affinity chromatography using a 50 mM potassium phosphate (pH 8.0) buffer containing 300 mM NaCl and 20 mM imidazole for loading and 250 mM imidazole for elution. ClyAwt for CD and single-molecule measurements was further purified following Eifler *et al*.^7^, by chromatography on hydroxyapatite (Biorad) at pH 6.8, where elution was conducted with a linear gradient from 20 to 250 mM potassium phosphate. All purification steps were performed in the presence of 2 mM β-mercaptoethanol or 2 mM dithiothreitol (DTT), resulting in fully reduced ClyA. ClyAwt for the cross-linking experiments was, after the nickel chelate affinity chromatography, buffer exchanged to 10 mM sodium phosphate (pH 7.4) via a HiPrep desalting 26/100 column (GE Healthcare), purified further by anion exchange chromatography on a HiPrep Q FF 16/100 (GE Healthcare) in 10 mM sodium phosphate (pH 7.4) using a gradient of 0–1 M NaCl for elution, and then passed over a Superdex 200 10/300 column (GE Healthcare) in standard buffer (50 mM sodium phosphate, 150 mM NaCl, pH 7.4). The ClyA variants for labelling were further purified after the nickel chelate affinity chromatography during the labelling process (see below). Purified protein was flash frozen in liquid nitrogen and stored at −80 °C after the addition of DTT to 10 mM and glycerol to 10% (v/v) or Tween 20 to 0.001% (w/v). The mass of all variants was confirmed by electrospray ionization mass spectrometry (MS). The concentration of unlabelled ClyA was determined via its absorbance at 280 nm using an extinction coefficient of 30,370 M^−1^ cm^−1^.

### Site-specific fluorescence labelling

Before labelling, DTT was added at a final concentration of 100 mM to the protein solution, to ensure complete reduction of all cysteine residues, and subsequently removed by passing the sample over a HiTrap Desalting column (GE Healthcare) in standard buffer. After concentrating the protein to 100–200 μM using a Vivaspin 10-kDa molecular weight cut-off centrifugal filter (Vivaproducts), Alexa Fluor 488 C5 maleimide (Invitrogen) dissolved in dimethyl sulfoxide was added at a molar ratio of 1:0.7 of dye:protein to label the first cysteine residue. As the thiol of Cys56 proved to be much more reactive than that of Cys252, sub-stoichiometric labelling yielded ClyA uniformly labelled at Cys56 with Alexa Fluor 488 (see below). The reaction was performed at 22 °C for 30 min and quenched by the addition of DTT (100 mM final concentration). Singly labelled protein was separated from unlabelled and doubly labelled protein by anion exchange chromatography, using a MonoQ 5/50 GL column (GE Healthcare) in 10 mM potassium phosphate (pH 7.4) and 10 mM NaCl, with an elution gradient of 10–300 mM NaCl. The protein was concentrated to 20–50 μM, and Alexa Fluor 594 C5 maleimide (Invitrogen) dissolved in dimethyl sulfoxide was added at four- to fivefold molar excess over protein. After incubation for 1–2 h at 22 °C, the reaction was again quenched by 100 mM DTT. Unreacted dye was removed by size-exclusion chromatography with a Superdex S75 10/300 column (GE Healthcare) in standard buffer. Site-specific labelling was confirmed by total mass analysis of the intact protein by electrospray ionization MS and analysis of tryptic fragments by matrix-assisted laser desorption ionization time-of-flight MS. The analysis confirmed uniform labelling of Cys56 with Alexa Fluor 488 and Cys252 with Alexa Fluor 594.

### Haemolysis kinetics

Kinetics of horse erythrocyte lysis by ClyAwt were measured as described previously^47^. Haemolysis was followed via the decrease in optical density at 650 nm using a Cary 100 spectrophotometer (Agilent) with a stirred cuvette. Horse erythrocytes (2 × 10^6^ ml^−1^, yielding an initial optical density of 0.75) were lysed by ClyA in PBS buffer (20 mM KH_2_PO_4_/K_2_HPO_4_, 150 mM NaCl, pH 7.3) at 37 °C. Haemolysis kinetics were evaluated by linearly fitting (i) the pre-transition baseline and (ii) the data points in the middle of the lysis reaction between 35% and 75% of the initial optical density. The lag phase of haemolysis was defined as the time point where the two linear fits intersected, and the maximum lysis rate was defined as the time derivative of the approximately linear decrease in optical density between 35% and 75% of the initial cellular density.

### Lifetimes, anisotropies and transfer efficiencies

Ensemble measurements of time-resolved fluorescence lifetime decays were carried out on a custom-built instrument^48^. For exciting Alexa 488 and 594, laser light from a white-light continuum source (SC-450AOTF, 20 MHz, Fianium) was passed through HQ470/40 (Chroma) and z582/15 (Semrock) interference filters, respectively. The *G*-factor was determined to be 0.92. Measurements were performed for ClyA in its monomeric form (50 nM ClyA56wt, 20 mM K_2_HPO_4_, 150 mM NaCl, 0.001% (w/v) Tween 20, 10 mM DTT) and in the pore complex (same buffer with 0.1% (w/v) DDM and 5 μM unlabelled ClyAwt after incubation for 1 h at 22 °C) at a concentration of 50 nM labelled protein. For both conditions, a donor-only labelled sample and one containing both donor and acceptor were measured. For each sample, the horizontally and vertically polarized fluorescence decays were measured for 5 min at a count rate of 4,000 s^−1^. For fluorescence lifetime analysis, the horizontally (*I*_h_(*t*)) and vertically (*I*_v_(*t*)) polarized decays were combined to *I*(*t*)=*I*_v_(*t*)+2*G I*_h_(*t*), to eliminate the anisotropy contribution, and fitted with 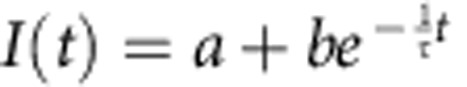, to obtain the fluorescence lifetime, *τ*. From the measurements of the donor lifetime in the presence (*τ*_DA_) and absence (*τ*_D_) of the acceptor dye, the transfer efficiency was calculated using 
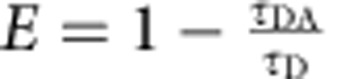
. For the anisotropy, the decays were combined to 
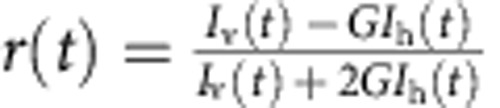
 and fitted with 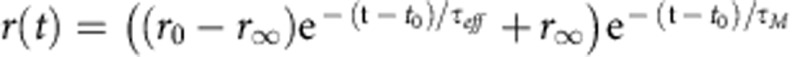 as described previously^48^,^49^. The steady-state anisotropy, *r*_ss_, was calculated from the integrated intensities, *I*_v,t_ and *I*_h,t_, according to 
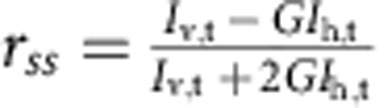
.

The four-channel setup of the single-molecule instruments permits the measurements of fluorescence lifetimes and polarization anisotropies directly from the single-molecule fluorescence data^50^. Subpopulation-specific fluorescence decay curves were generated from fluorescence bursts selected, as described in the Methods section, and located in a specified transfer efficiency range. The signal from the horizontally (*I*_h_(*t*)) and vertically (*I*_v_(*t*)) polarized fluorescence decays were combined to *I*(*t*)=*I*_v_(*t*)+2*GI*_h_(*t*) (with *G*=1.245) to eliminate the anisotropy contribution and fitted with 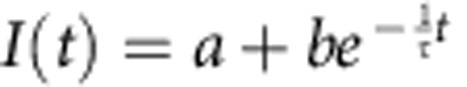 to obtain the fluorescence lifetime, *τ*. For *τ* of the donor in the presence of the acceptor, the sum of two exponential decays was fitted to the decay curve and the lifetime was taken as the average of the two lifetimes weighted by their amplitudes. To determine absolute anisotropy values from the single-molecule data, corrections for the geometry of the objective have to be introduced^51^. The *L*-factors determined for our instrument were *L*1=0.098 and *L*2=0.094 for excitation with 488 nm, and *L*1=0.111 and *L*2=0.058 for excitation with 585 nm. The steady-state anisotropy was then calculated according to 
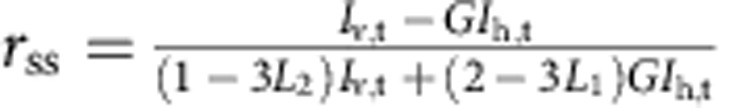
.

### CD spectroscopy

The monomer-to-pore transition of ClyAwt monomer (in the range of 2.8–9 μM) in PBS buffer (pH 7.3) at 22 °C was initiated by the addition of DDM (final concentration: 0.1% w/v) and followed via the changes in ellipticity at 225 and 280 nm with a temperature-controlled Jasco J715 CD spectrometer. At 225 nm, the rapid first phase of the conformational transition was additionally measured with a PiStar stopped-flow spectrometer (Applied Photophysics). Kinetic traces obtained by manual mixing and stopped-flow measurements were combined and converted to mean residue ellipticity. Far-ultraviolet CD spectra of 0.3 mg ml^−1^ ClyAwt monomer, protomers in pores (incubated for 2 h in 0.1% DDM) and unfolded protein (incubated for 2 h in 5 M GdmCl) were obtained in PBS buffer (pH 7.3) at 22 °C using a Jasco J715 CD spectrometer with a 0.1-cm cuvette. The far-ultraviolet CD spectrum of the ClyA intermediate 40 s after the initiation of the conformational transition with 0.1% DDM was reconstructed from kinetic traces recorded at constant wavelength in the range of 203–250 nm. For the comparison of the kinetics of ClyAwt and unlabelled ClyA with amino acid replacements Q56C and E252C, the initial phases of both kinetics were measured at 225 nm in PBS buffer (pH 7.3) at 22 °C with 0.1% (w/v) DDM and 2 mM DTT.

### Cross-linking analysis of ClyA pore formation

Cross-linking of ClyA oligomers was achieved by ‘photo-induced cross-linking of unmodified proteins’^22^,^52^. Pore formation was started by incubating 500 nM ClyAwt in standard buffer containing 0.1% (w/v) DDM and 0.001% (w/v) Tween 20 at 22 °C. At various times, 9 μl samples were withdrawn, mixed with 1 μl 0.5 mM tris-bipyridylruthenium(II) and 10 mM ammonium persulfate (xlink mix) in a thin-walled PCR tube (Sarsted), and illuminated with 488-nm laser light (FCD488-010, JDSU) at 20 mW for 1.5 s. The cross-linking reaction and pore formation were then stopped immediately by the addition of 5 μl NuPAGE 4 × LDS loading buffer (Invitrogen) containing 150 mM DTT. After heating the samples for 10 min at 75 °C, they were run on NuPAGE 3%–8% tris-acetate polyacrylamide gels (Invitrogen). For quantitative analysis, the gels were stained according to the fast Fairbanks protocol^53^ and scanned with an Odyssey scanner (Li-Cor) using the built-in 685 nm laser. Band intensities were integrated using Mathematica (Wolfram). For qualitative analysis, the gels were subsequently stained using the ProteoSilver Silver Stain Kit (Sigma). The apparent molecular mass of the bands was estimated from their retardation factors relative to the marker bands (PageRuler Prestained Protein Ladder, Thermo Scientific).

### Single-molecule measurements

Single-molecule measurements were performed on two modified Micro Time 200 confocal instruments (Picoquant) and a custom-built four-channel instrument. Excitation light was provided by a pulsed diode laser (LDH-D-C-485, PicoQuant) via an HC Triple laser beam splitter BS R405/488/594 (Semrock) at a power of 100 μW at 20 MHz for the donor dye and white light continuum sources (SC-450AOTF, SC-400-6 or SC-450-6, Fianium) filtered through a z582/15 band-pass filter (Chroma) and a HC 720/SP infrared filter (Semrock) at a power of 50 μW at 20 MHz for the acceptor. The sample was illuminated and epifluorescence collected with an UplanApo 60 × /1.20 W objective (Olympus). Fluorescence light was passed through a 100 μm pinhole, split first by polarization (polarization cube, PicoQuant) and then by colour (595 DCXR (Chroma)), and detected by avalanche photodiodes (MPD 100ct (Micro Photon Devices), SPCM-AQR-13 (PerkinElmer Optoelectronics) and *τ*-SPAD (PicoQuant)) after passing additional filters (ET 525/50 (Chroma) for donor-dye detection and HQ 650/100 (Chroma) for acceptor-dye detection). Arrival times of detected photons were recorded with a time interval analyser and time-correlated single-photon counting system (HydraHarp 400 (PicoQuant)) at a resolution of 16 ps. All measurements were done with pulsed interleaved excitation^54^. For some kinetic measurements, a second LDH-D-C-485 diode laser, delayed relative to the first one, was included in the setup to increase the FRET signal collected per time relative to the signal from acceptor direct excitation. All measurements were done at 22 °C with ~100 pM labelled protein in single-molecule buffer (20 mM potassium phosphate (pH 7.3) containing 150 mM NaCl, 0.001% (w/v) Tween-20, 10 mM DTT and other additives as indicated in the main text or below). The concentration of Tween-20 used here does not trigger the conformational change of ClyA (Fig. 1b) and merely serves to minimize the loss of labelled protein to vessel surfaces.

The initial phase of the ClyA protomer formation was measured in a microfluidic continuous-flow mixing device specifically developed for single-molecule detection^20^,^23^. The centre channel was pretreated with BSA and PLL-PEG^20^ to reduce ClyA adhesion to the channel walls, and filled with sample containing 1.6–4.3 nM labelled protein in single-molecule buffer containing 100 mM DTT. The buffer channels were filled with the same buffer containing no DTT but 0.111% (w/v) DDM. The applied pressures were 2.33 and 2.46 psi at the centre and buffer channels, respectively, to achieve a mixing ratio of 1:9 and an average flow velocity of 1 mm s^−1^. Manual-mixing experiments were done in glass cuvettes coated with poly(L-lysine)-*graft*-poly(ethylene glycol) co-polymer (PLL(20)-g[3.5]-PEG(2), Susos). To that end, cuvettes were treated with oxygen plasma for 30 s in a FEMTO plasma cleaner (Diener) at 25% power, then incubated with 0.1 mg ml^−1^ PLL-PEG in double-distilled water for at least 10 min. For the measurements, 25 μl of 200 pM labelled ClyA in single-molecule buffer containing 20 mM DTT were mixed in a sample cell with 25 μl of the same buffer containing no DTT but 0.2% (w/v) DDM. To improve the signal-to-noise ratio, the measurements were repeated several times and the results combined. To observe the pore formation kinetics by manual mixing, unlabelled ClyAwt was added to increase the overall protein concentration. Reference measurements for the protomer and monomer state were done under the same conditions in the absence of ClyAwt (and DDM in case of the monomer). The final DDM concentration of 0.1% (w/v) is above the critical micelle concentration (0.009% (w/v) in water^55^).

### Single-molecule data reduction

Photon time series were first time-gated to select the emission resulting from the different wavelengths for donor and acceptor excitation. Emission after donor excitation was corrected for the different quantum yields of the dyes, the different detection efficiencies of the detectors, cross-talk and acceptor direct excitation, and background^56^. Fluorescence bursts resulting from single molecules were identified as contiguous intervals of emission with interphoton times^57^ <0.1 ms (see Supplementary Table 2 for minimum and maximum numbers of photons per burst). From the identified bursts were selected those which showed emission after acceptor direct excitation^54^, with a stoichiometry ratio (*S*) of <0.7. *S* is defined as 
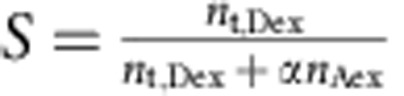
, with *n*_t,Dex_ being the total number of photons after donor excitation, *n*_Aex_ the number of photons after acceptor direct excitation and *α*, a factor chosen in order that the main FRET population is at *S*=0.5. To eliminate fluorescence bursts during which acceptor bleaching occurs, only bursts with a difference of <0.2 ms in the mean arrival times of the photons in the channels after donor and acceptor excitation, respectively, were used. The resulting bursts were binned in a histogram according to transfer efficiency (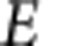), calculated for each burst from the number of photons from the corrected donor (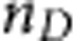) and acceptor photon counts (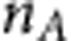) after donor excitation according to 
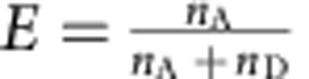
. To improve the signal-to-noise ratio for kinetic measurements using manual mixing, the corresponding transfer efficiency histograms from several repeat measurements of the same length were added up. Numbers of measurements and window sizes for the different data sets are listed in Supplementary Table 2.

### Transfer efficiency histogram analysis

To identify populations from the transfer efficiency histograms, peaks in the histograms were approximated with Gaussian (*G*) and a four-parameter log-normal (*L*) peak functions for symmetric (monomer, protomer and pore) and asymmetric (intermediate) peaks, respectively: 
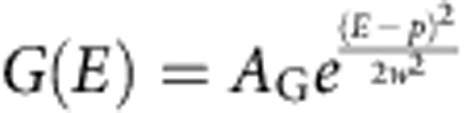
 and 
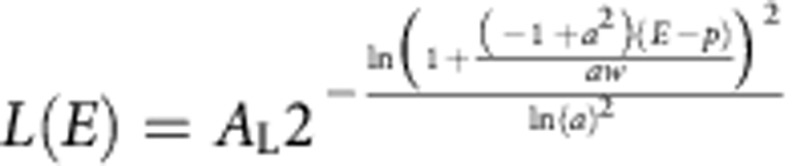
, with *E* being the transfer efficiency, *A* the peak amplitude, *p* the peak position, *w* the peak width and *a* the asymmetry of the peak^24^,^25^,^26^. For fitting more than one peak, the histogram was analysed with a sum of Gaussian and log-normal functions. Whenever several histograms containing peaks from the same species were analysed, the peaks were described with a global fit over all histograms, with *p*, *w* and *a* (where applicable) for each species as shared parameters. The uncertainty in transfer efficiency was estimated from the s.d. of six different measurements of the monomer collected over the course of 4 years. For the conversion of transfer efficiency to distances, a Förster radius of 5.4 nm was used^58^.

Transfer efficiency histogram time series obtained in the microfluidic mixer could be described using a global fit with three species, one log-normal peak function for the intermediate and a Gaussian each for the monomer and protomer, over all histograms, as the three peaks are suitably separated. As the monomer population is not observed to a sufficient extent in the manual mixing experiments, for the global fit, *p* and *w* of the monomer were fixed to the values from measurements containing only the monomer. In the pore formation measurements, the peak separation between protomer and oligomers is less pronounced. Except for the measurements at 5 nM total ClyA concentration, where the protomer species was sufficiently populated during the kinetics, *p* and *w* for the protomer were fixed based on independent measurements at sub-nanomolar concentrations of labelled ClyA, where no pores are formed. Selected parts of the beginning and end of the pore formation kinetics showing clear peaks for the intermediate and the pore were then fitted with the parameters for monomer and protomer fixed to obtain the parameters for intermediate and pore. The peak parameters from this procedure were then used to fit the histograms by varying the amplitudes, either individually for each histogram, or globally, as constrained by the respective kinetic models, to obtain the fractions of the different species (see below and Supplementary Fig. 2).

### Global analysis of FRET efficiency histogram time series

For the microfluidic measurements, the conversion of the position in the observation channel to the time after mixing was done with the help of finite-element calculations as described previously^59^. Calculations were performed using the diffusion coefficients of the ClyA monomer and protomer as determined by 2f-FCS. For the manual mixing experiments, the time for each histogram was defined as the middle of the time window with an offset of 10 s, to account for the time between mixing and the start of data acquisition. Kinetic models were fit directly to the resulting time series of transfer efficiency histograms using the following procedure (implemented in Mathematica, Wolfram Research). The systems of differential equations describing the different kinetic models were solved numerically and the concentrations of all species were calculated for the times corresponding to each histogram. To compare these species fractions with the histograms, the histogram peaks were then reconstructed using the peak parameters as determined above and calculating the amplitudes of each peak from the fraction of the corresponding species, *f*, (that is, the normalized concentrations), 
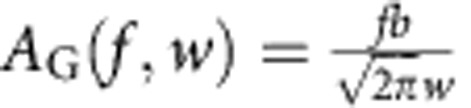
, and the empirical function 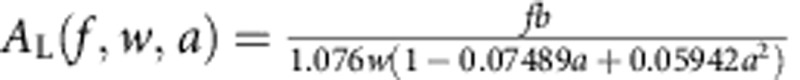, where *b* is the histogram bin width and *w* and *a* the peak parameters described above. The sum of the reconstructed peaks was then subtracted from the measured histogram (normalized by the number of bursts), and these residuals from all histograms involved in the global fit were minimized to obtain the rate coefficients of the kinetic model (see Supplementary Fig. 2 for an illustration of the process). For fitting the rates of pore formation, all histograms from all data sets were used, including the ones at 100 pM, where no pore formation is visible. To be able to apply the same model including a pore population for these histograms, the mean values of the *p* and *w* parameters of the other data sets were used as parameters of the pore peak. In this fit, only the rate coefficients of the oligomerization reactions were free parameters; those of protomer formation were fixed to the values obtained as described above. As a measure for the quality of the fits, the sum of the squared residuals was calculated for each histogram as 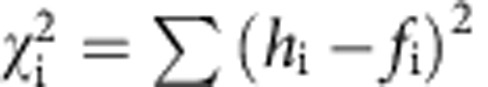 and the whole data set as 
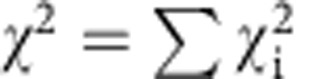
, with *h*_*i*_ being the histogram value at transfer efficiency bin *i*, and *f*_*i*_ the value of the fit function at this point. The uncertainties of the fitted rate coefficients were obtained as described below.

### Uncertainty estimation of the rate coefficients obtained from the fits

To estimate the uncertainty of the rate coefficients that result from our procedure of globally fitting the transfer efficiency histogram time series (see ‘Global analysis of FRET efficiency histogram time series’ in Methods), we took the approach of varying the model parameters that are constrained in the global fitting procedure to the values determined independently (see ‘Transfer efficiency histogram analysis’ in Methods). For the fit describing protomer formation, we repeated the fit 1,000 times and varied in every iteration two randomly chosen peak parameters (positions, widths and asymmetries) describing the histograms, one for the histograms from the microfluidic and one for the histograms from the manual mixing experiments. As the peak parameters are not independent, the remaining peak parameters were determined by fitting the histograms as described in Methods (Transfer efficiency histogram analysis), with the varied parameters fixed to the new values. The variations of the parameters were drawn from normal distributions. In case of the peak positions, the distribution had an s.d. of 0.03 (see Methods and Supplementary Table 1); in case of the other parameters, the s.d. was chosen to be 5% of the parameter’s value. For the fit describing pore formation, the peak parameters and the rate coefficients for protomer formation obtained at 100 pM ClyA (where no pores are formed) were used. To estimate the uncertainty in the rate coefficients of assembly, we varied the rate coefficients within the distributions obtained for protomer formation. As the individual rate coefficients are not independent, the respective combinations of *k*_1_ to *k*_4_ resulting from the individual fits were used. The uncertainties in the rate coefficients are given as the weighted means and s.d. The rate coefficients were weighted by the inverse *χ*^2^-value of the respective fit.

### Bootstrap analysis of the protomer formation data set

Model performance and fit parameter variation was tested with bootstrap analysis on the level of the fluorescent bursts data. New artificial data sets were created on the basis of the original lists of fluorescence bursts by randomly drawing with replacement, and each new data set was subjected to the complete fitting process (see Methods and Supplementary Fig. 2) with both the on- and the off-pathway model. To introduce additional variation, noise was added to various degrees to the transfer efficiency of each burst. The added noise was taken from normal distributions with mean 0 and s.d. of 0.01, 0.02, 0.05 and 0.1. Each procedure was repeated 50 times, and the total *χ*^2^-value, the mean and s.d. of the fit parameters were calculated to get an estimate of their variation and the robustness of the fitting procedure (Supplementary Fig. 4).

### Fluorescence correlation spectroscopy

The absolute diffusion coefficients of several conformational states of ClyA were determined by 2f-FCS^21^ at 22 °C on a modified Micro Time 200 (PicoQuant) as for the single-molecule measurements with the following changes. The sample was excited alternatingly with two orthogonally polarized diode lasers (LDH-D-C-485, PicoQuant) at a power of 30 μW at 40 MHz each. Before the objective, a differential interference contrast prism (U-DICR, Olympus) was inserted into the beam path to split the two polarizations into two foci. The inter-focus distance was determined following a published procedure, using four samples with different diffusion behaviour (Oregon Green, denatured AlexaFluor488-labelled CspTmC67, hCypV2C and monomeric GroEL-single ring), whose value of *R*_S_ had been determined independently by dynamic light scattering^60^. Samples for labelled ClyA in the monomer, protomer and pore state were prepared as described for the single-molecule FRET experiments, using 100 pM labelled protein for the protomer measurements, 1 nM for the monomer and 1.3–1.7 nM labelled ClyA in the presence of 5 nM to 5 μM unlabelled ClyA for the pore measurements. To eliminate the contribution of occasional larger aggregates, the photon time traces were binned in 1-ms bins and photons from bins containing more than 1,000 photons were, together with the photons from directly adjacent bins, removed from the trace. Refractive indices of buffers were measured with an Abbe refractometer (Krüss) and viscosities determined with a digital viscometer (DV-I+, Brookfield Engineering) with a CP40 spindle at 100 r.p.m. The fitting procedure of the data sets and the method to model the 2f-FCS curves according to the kinetic model are presented below. The hydrodynamic radii given in the main text are means and s.d. of three measurements. To compare how the populations of oligomeric intermediates predicted by our model agree with the 2f-FCS data, especially at concentrations where only few complete pores are formed, we quantified the increase in ‹*R*_S_› using the concentrations for all species obtained from the model (see below). The diffusion coefficients of monomer, protomer and complete pore were determined individually by 2f-FCS and the diffusion coefficient of the molten-globule-like intermediate was observed to be very similar to that of the protomer (Supplementary Fig. 6). The resulting value of *R*_S_ for the pore was 8.0±0.3 nm, close to the 6.7 nm calculated with HydroPro^61^ based on the crystal structure^17^. (The remaining difference is most probably due to detergent bound to the pore.) The values of *R*_S_ for the oligomeric intermediates were calculated with HydroPro based on the pore crystal structure with the corresponding numbers of subunits removed and scaled slightly to account for bound detergent (Supplementary Fig. 10). Subpopulation-specific FCS^62^ curves for ClyA with cysteine residues 87 and 285 replaced by alanine in 0.1% DDM were calculated from fluorescence bursts from either the intermediate or the protomer, chosen based on their transfer efficiencies and stoichiometry ratios (Supplementary Fig. 6a).

### 2f-FCS fitting procedure and modelling

The four correlation curves resulting from a 2f-FCS measurement (the autocorrelation of each focus and the cross-correlations between the foci) were fit globally with the model of Dertinger *et al*.^21^ with the addition of a triplet state component:


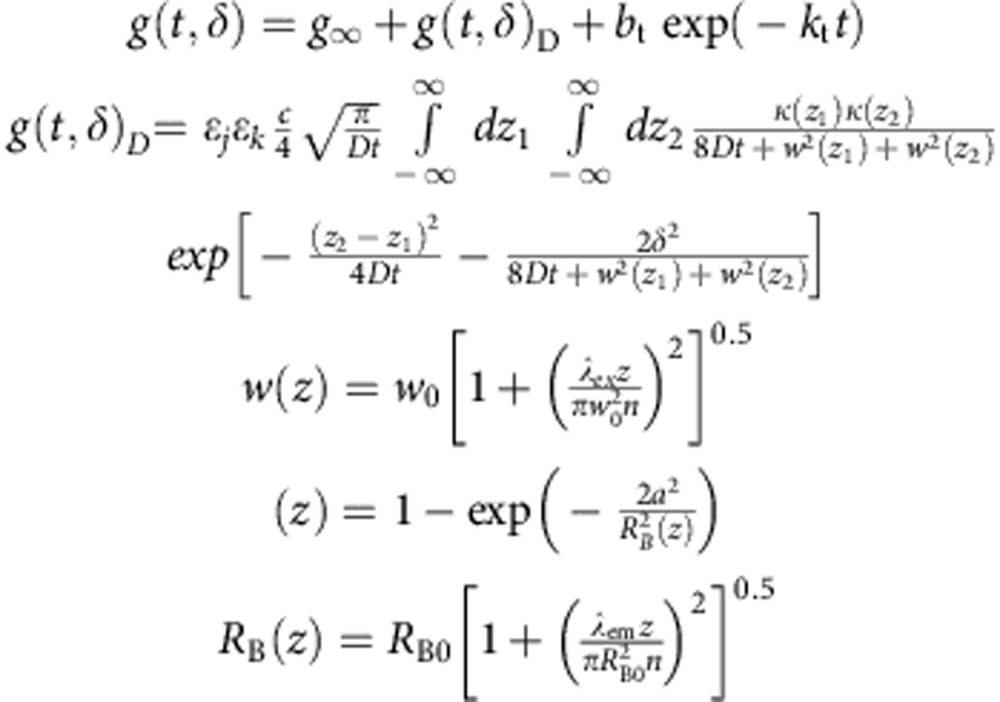


The functions *w*(*z*), *κ*(*z*) and *R*(*z*) describe the molecule detection function as described in the original paper^21^, *λ*_ex_ is the excitation wavelength, *λ*_em_ the emission wavelength, *a* the pinhole radius divided by the magnification, *w*_0_ and *R*_B0_ are the geometry parameters of the molecule detection function, *n* the refractive index of the solution, *δ* the distance between the foci, *D* the translational diffusion coefficient, *b*_t_ the triplet amplitude, *k*_t_ the triplet rate, *ε*_*j*_ and *ε*_*k*_ the overall detection efficiency of one focus *j* and *k*, respectively, and *c* the concentration of the labelled species. For the autocorrelations, *δ* is set to zero, *ε*_*j*_ to *ε*_*k*_ for focus *k* and *ε*_*k*_ to *ε*_*j*_ for focus *j*. The inter-focal distance was 437 nm, (ref. 60) *λ*_ex_ was 485 nm, *λ*_em_ 515 nm, the pinhole radius 75 μM, the magnification 60 × and the refractive index for the buffer used was 1.335. The global fit parameters were 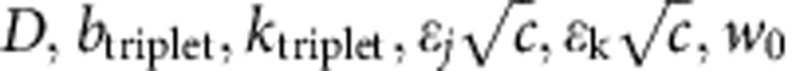, and *R*_B0_; *g*_*∞*_ was fitted for each correlation separately. One diffusive species and one triplet state were sufficient to fit every data set. The kinetic measurements (*cf*. Fig. 6) were first fit over the entire time range, to determine the geometry parameters, *w*_0_ and *R*_B0_. Next, the measurements were split into 5-min segments and each segment was fit separately, with the geometry parameters fixed. The diffusion coefficients, *D*, were converted to Stokes radii, *R*_S_, using the Stokes–Einstein equation, 
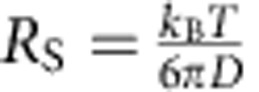
, with *k*_B_ being the Boltzmann constant, *T* the temperature and *η* the solvent viscosity (0.95 mPa s for the buffer without DDM, 0.97 mPa s for the buffer with 0.1% (w/v) DDM and 1.35 mPa s for the buffer containing 5 M guanidinium chloride). For comparison, diffusion coefficients and Stokes radii were calculated for the monomer, protomer (using one pore subunit) and the complete pore based on the crystal structures (PDB codes 1QOY^18^ and 2WCD^17^) using HydroPro^61^.

To test which population predictions of the kinetic assembly models used to fit the time series of transfer efficiency histograms (Fig. 4 and Supplementary Fig. 9) were compatible with the kinetic 2f-FCS measurements, the correlation curves, *g*(*t*, *δ*), were reconstructed according to the concentration distributions of the oligomeric species predicted from the fits, then fitted similar to the measured 2f-FCS data and the resulting diffusion coefficients were compared. The parameters necessary to reconstruct *g*(*t*, *δ*) were obtained the following way. The triplet parameters, the geometry parameters and the *g*_∞_ values were taken from the fits to the measurements. The diffusion coefficients for monomer, protomer and complete pore were measured, the one for the intermediate species was set equal to the protomer and those for the oligomeric species were calculated. To this end, HydroPro^61^ was used to calculate diffusion coefficients for all oligomer sizes using the pore structure^17^ and deleting one subunit after the other. As expected from Stokes’ law, the resulting calculated diffusion coefficients scale approximately with *n*^−1/3^, where *n* is the number of subunits. Accordingly, we interpolated between the measured values of protomer and pore with the same functional form, to estimate the diffusion coefficients of the intermediate oligomers (Supplementary Fig. 10). The overall detection efficiency of a focus was taken as the apparent brightness of the species, calculated as *b*_app_(‹*E*›, *m*, *f*_l_)=(1–‹*E*›)‹*n*›_dyes_(*m*, *f*_l_), where ‹*E*› is the mean transfer efficiency of the species, *m* the number of monomers, *f*_l_ the fraction of labelled to unlabelled protein in the measurement and ‹*n*›_dyes_(*m*, *f*_l_) is the average number of fluorophores in that species. ‹*n*›_dyes_ was set to 1 for all monomeric species and calculated for the multimeric species assuming a binomial distribution, 
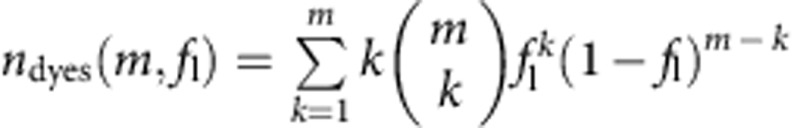
. For the differences in detection efficiencies of the two foci, the ratio 
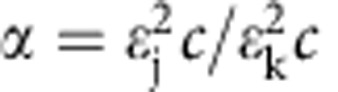
 was determined from the fits to the measurements. Thus, for the reconstruction, 
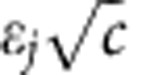
 and 
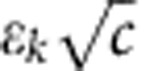
 were replaced by 
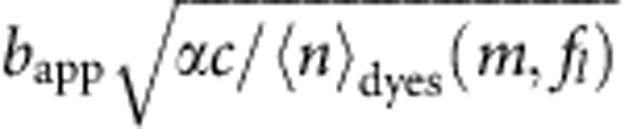
 and 
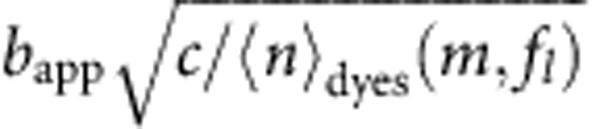
, respectively. The reconstruction was done using 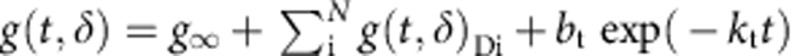 with the sum over the number of all species in the model. These reconstructed curves were fit in the same way as the measurements (that is, with one species) and the resulting diffusion coefficients compared with the one of the measurement (Fig. 6).

## Author contributions

B.S. and R.G. designed the research. S.B. and D.R. prepared protein samples and performed the measurements. S.B., D.R., D.N., R.G. and B.S. analysed and interpreted the data. D.N. developed instrumentation, data analysis tools and assisted with data analysis. B.W. provided the microfluidic mixing devices and performed finite-element calculations. S.B. and B.S. wrote the paper with the help of the other authors.

## Additional information

**How to cite this article:** Benke, S. *et al*. The assembly dynamics of the cytolytic pore toxin ClyA. *Nat. Commun.* 6:6198 doi: 10.1038/ncomms7198 (2015).

## Supplementary Material

Supplementary Information Supplementary Figures 1-11, Supplementary Tables 1-2 and Supplementary References
