# Erratum: The assembly dynamics of the cytolytic pore toxin ClyA

**DOI:** 10.1038/ncomms10650

**Published:** 2016-02-01

**Authors:** Stephan Benke, Daniel Roderer, Bengt Wunderlich, Daniel Nettels, Rudi Glockshuber, Benjamin Schuler

Nature Communications 6: Article number: 6198 10.1038/ncomms7198 (2015); Published: 02052015; Updated: 02012016

In Figure 2b of this article, the sets of arrows connecting 'I' and 'M' in the kinetic scheme are inverted. The correct version of Fig. 2 appears below.


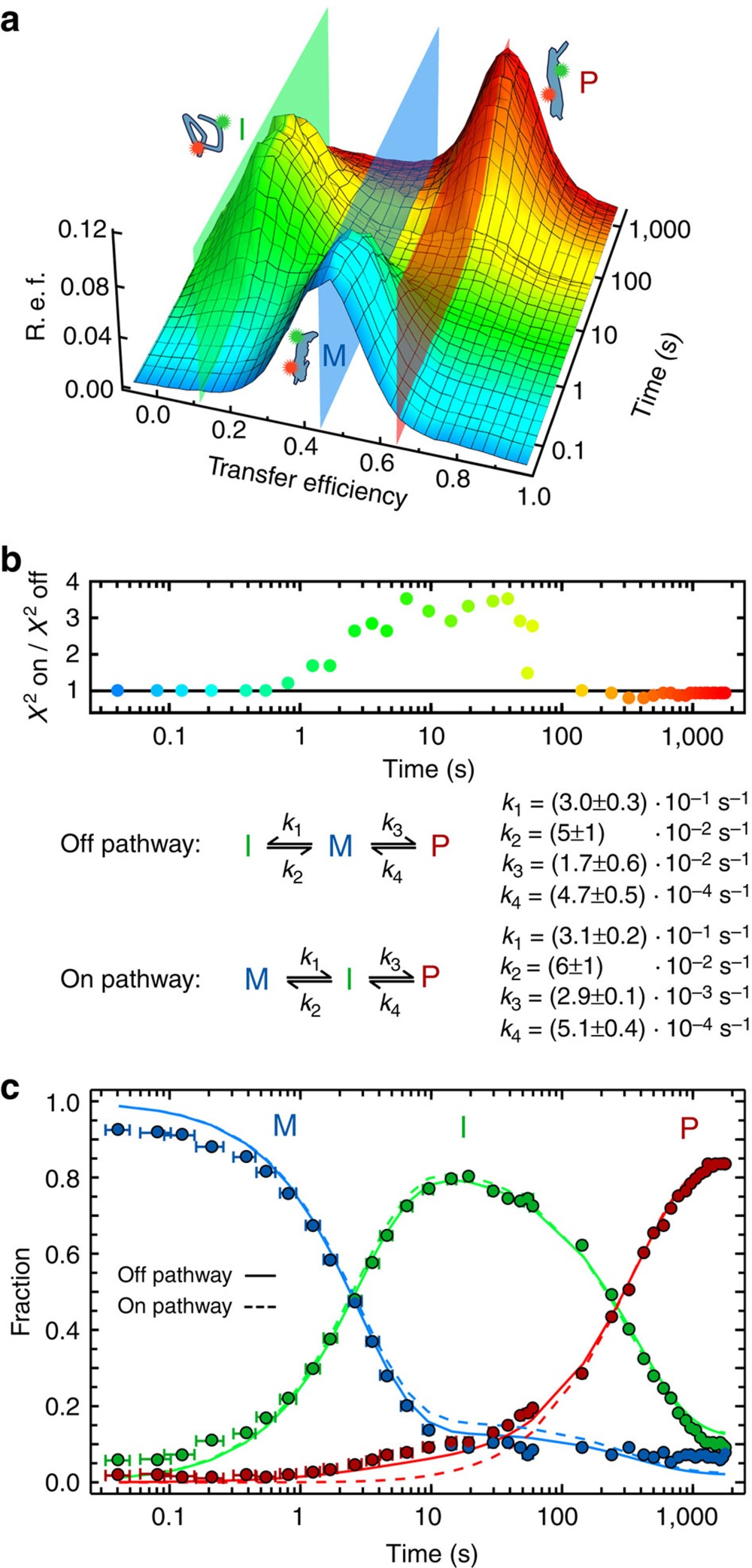


**Figure 2 |**

