# Supplementary material for: The assembly dynamics of the cytolytic pore toxin ClyA
Source: Nat Commun. 2015 Feb 5;6:6198. doi: 10.1038/ncomms7198 (PMC4347018; doi:10.1038/ncomms7198)
Supplement: Supplementary Information — Supplementary Figures 1-11, Supplementary Tables 1-2 and Supplementary References [file ncomms7198-s1.pdf]

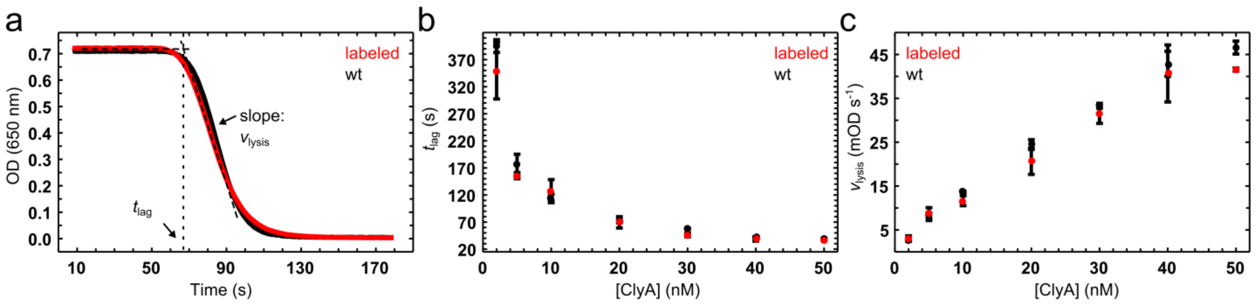

Supplementary Fig. 1. Haemolytic activity of fluorescently labelled ClyA. Horse erythrocytes were lysed with different concentrations of unlabelled wild-type protein (ClyAwt) and labelled ClyA to compare their activities. (**a**) Representative kinetic traces of lysis experiments with ClyAwt and labelled ClyA at identical protein concentrations of 20 nM. The maximum lysis rate (v_{lysis}) is approximated as the slope of the curve at the inflexion point, the lag time (t_{lag}) as the intersection of the resulting line with this slope and the extrapolated pre-lysis baseline. (**b**) Lag times for the lysis reaction at different concentrations of unlabelled and labelled ClyA. (**c**) Maximum lysis rates at different ClyA concentrations. Points and error bars correspond to means and standard deviations of three measurements.

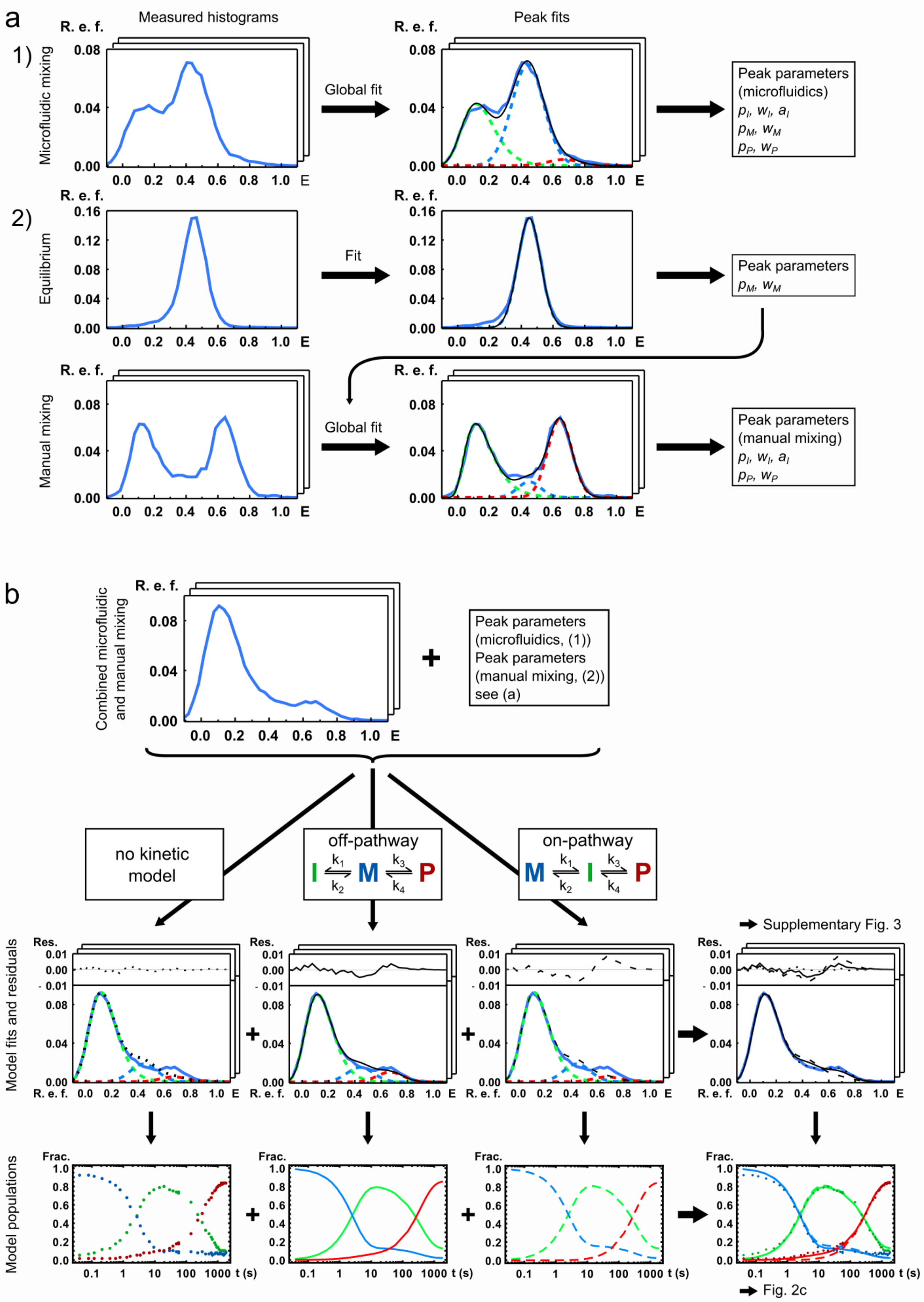
a
Measured histograms
Peak fits
1) Microfluidic mixing
R. e. f.
Global fit
Peak parameters (microfluidics) p_I, w_I, a_I p_M, w_M p_P, w_P
2) Equilibrium
Fit
Peak parameters p_M, w_M
Manual mixing
Global fit
Peak parameters (manual mixing) p_I, w_I, a_I p_P, w_P
b
Combined microfluidic and manual mixing
Peak parameters (microfluidics, (1)) Peak parameters (manual mixing, (2)) see (a)
no kinetic model
off-pathway
I ⇌ M ⇌ P (k_1, k_2, k_3, k_4)
on-pathway
M ⇌ I ⇌ P (k_1, k_2, k_3, k_4)
Supplementary Fig. 3
Model fits and residuals
Res.
Model populations
Frac.
t (s)
Fig. 2c

Supplementary Fig. 2. Illustration of the fitting procedure of the single molecule FRET data shown for the protomer formation dataset. (**a**) Determination of the peak parameters (position *p*, width *w*, and asymmetry *a*, but not the amplitude) of the functions used to fit the transfer efficiency histogram peaks (see also Methods section). Gaussian distributions and a 4-parameter log-normal distribution functions are used to empirically describe the monomer (M), intermediate (I), and protomer (P) populations in the histograms. 1) In the case of the data acquired in the microfluidic mixer, the parameters are determined by a global fit where the peak parameters (with the exception of the amplitudes) are shared for all histograms. 2) For the data from the manual mixing experiments, the parameters of the monomer population had to be determined from separate equilibrium measurements in the absence of DDM (owing to its low population in the kinetics at longer times) and were then constrained to these values in the global fit of all histograms. (**b**) Procedure to fit the kinetic models to the histograms. Using the peak parameters determined in (a), the histograms are fitted by using either free and independent peak amplitudes for each histogram (no kinetic model), or by having the amplitudes determined by a kinetic model. The rate coefficients that provide the best global fit can then be used to reconstruct the histograms based on the resulting peak amplitudes as a function of time. The reconstructed histograms are compared to the measured ones, yielding the residuals shown on top of the histograms (see also Supplementary Fig. 3). The peak areas for each histogram are then used to generate the time course of the relative populations, which is shown in Fig. 2c and Supplementary Figs. 8 and 9. The sums of the squared residuals for each histogram were used to compare the fit quality of the models (Fig. 2b). R. e. f.: relative event frequency, E: transfer efficiency, Res.: residuals, Frac.: fraction.

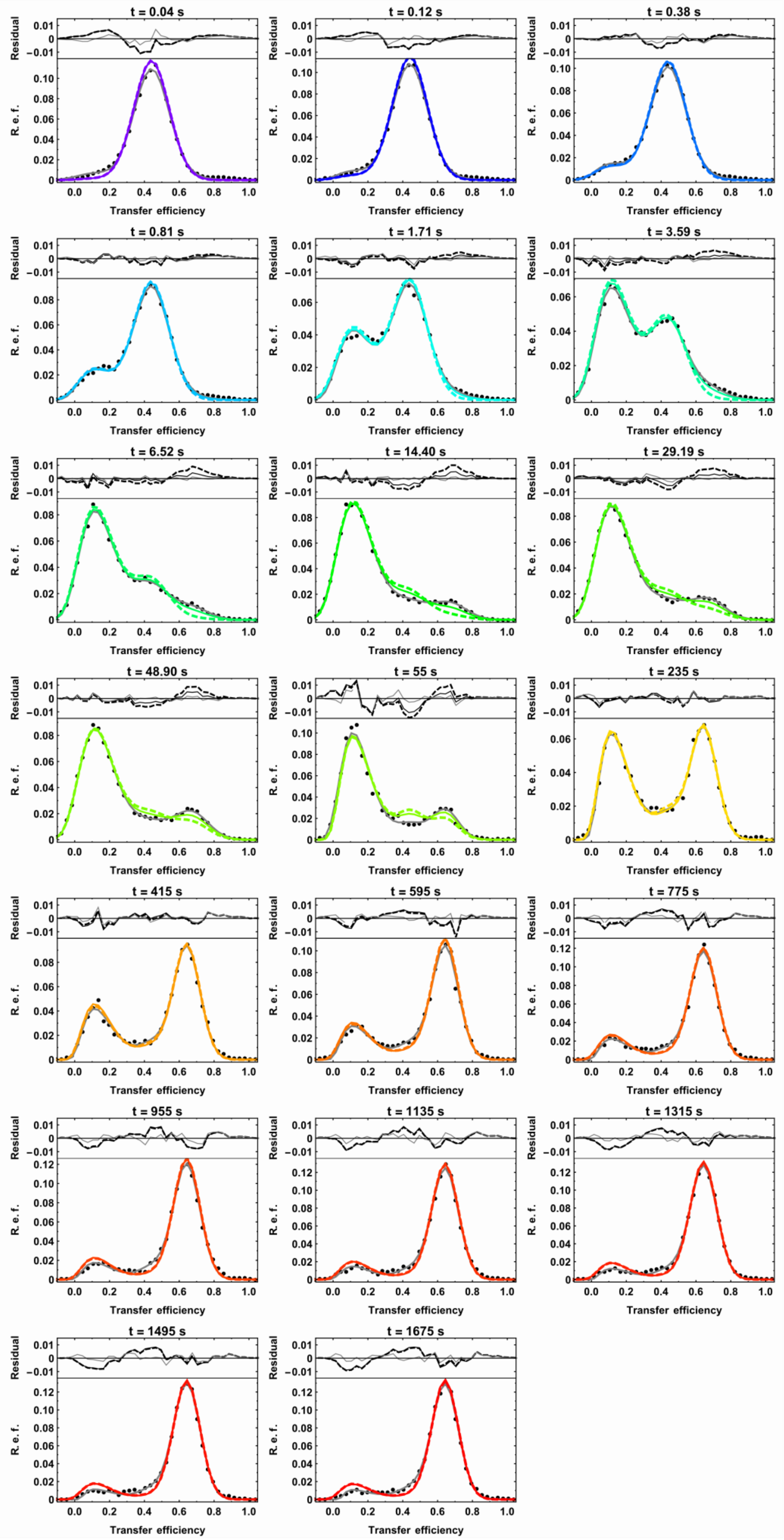
t = 0.04 s
t = 0.12 s
t = 0.38 s
t = 0.81 s
t = 1.71 s
t = 3.59 s
t = 6.52 s
t = 14.40 s
t = 29.19 s
t = 48.90 s
t = 55 s
t = 235 s
t = 415 s
t = 595 s
t = 775 s
t = 955 s
t = 1135 s
t = 1315 s
t = 1495 s
t = 1675 s
Residual
R. e. f.
Transfer efficiency

Supplementary Fig. 3. Comparison of measured and reconstructed transfer efficiency histograms for ClyA protomer formation based on the global fits. The fit of the two models and the corresponding residuals are illustrated for every other transfer efficiency histogram of the time series. The measured histograms (black points) are shown together with the reconstructions of the two model fits, the off-pathway model (solid coloured line), the on-pathway model (dashed coloured line), and the fit with unconstrained amplitudes (black line). The colour code is the same as in Fig. 2a. On top of each panel, the residuals are shown for the off-pathway model (solid black line), the on-pathway model (dashed line), and the fit with unconstrained amplitudes (solid grey line). See Methods for details and Supplementary Fig. 2 for an illustration of the fitting procedure. At the beginning and the end of the kinetics, the two kinetic models do not differ in fit quality. They only differ between 1 s and 100 s, with the off-pathway model providing the better fit (see Fig. 2b and also Supplementary Fig. 4). Note that the histogram at 55 s is the first one from the manual mixing experiments, resulting in a slightly more pronounced deviation of both fits at this transition between the microfluidic and manual mixing measurements. R. e. f.: Relative event frequency.

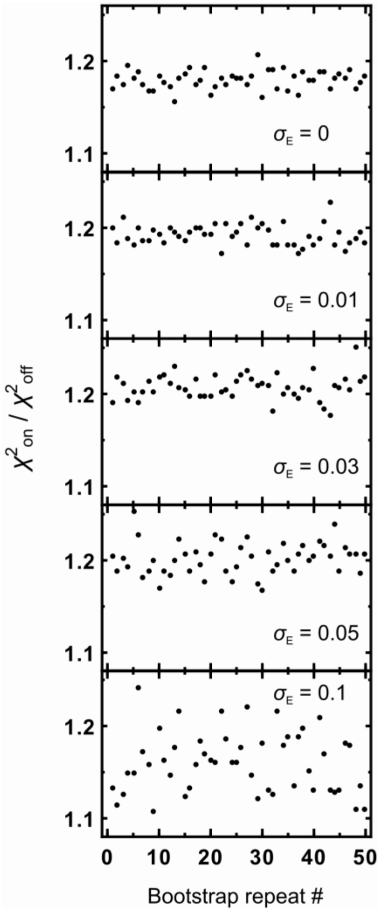

Supplementary Fig. 4. The better fit of the off-pathway model is robust. The performance of the off-pathway and on-pathway models was tested with bootstrap analysis on the level of the fluorescent bursts from which the transfer efficiency histograms were built, with various degrees of normal distributed noise (standard deviation σ_E) added to the transfer efficiency of each burst (see Methods). Displayed is the χ^2 (total χ^2 of the fit to all histograms, i.e. the sum of the χ^2 shown in Fig. 2b) ratio of the on- (χ^2_{on}) and the off-pathway (χ^2_{off}) model fits for 50 repeats of the different bootstrap procedures. Note that in all cases the off-pathway model performs better than the on-pathway model, even with a large amount of added noise. The variations in the fit parameters resulting from these perturbations are < 5 % for the peak parameters and < 15% for the rate coefficients.

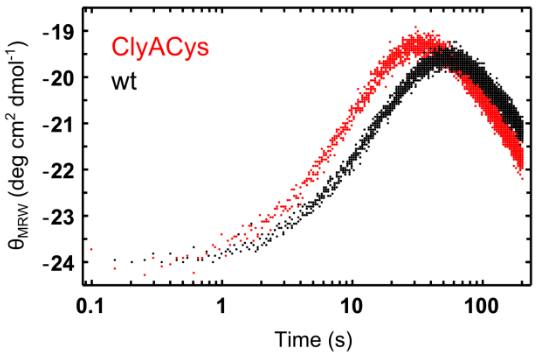

Supplementary Fig. 5. Influence of cysteine residues introduced for labelling on the kinetics of the conformational changes of ClyA. Stopped-flow measurements of the CD signal at 225 nm of unlabelled ClyA with Cysteine residues at positions 56 and 252 (4 μM, red) and ClyAwt (5 μM, black) in 2 mM DTT after triggering the conformational change by mixing with DDM (final concentration 0.1 % (w/v)). The time of the maximum in Θ_{MRW} is shifted only slightly from ~50 s to ~35 s by the introduction of the cysteine residues at positions 56 and 252.

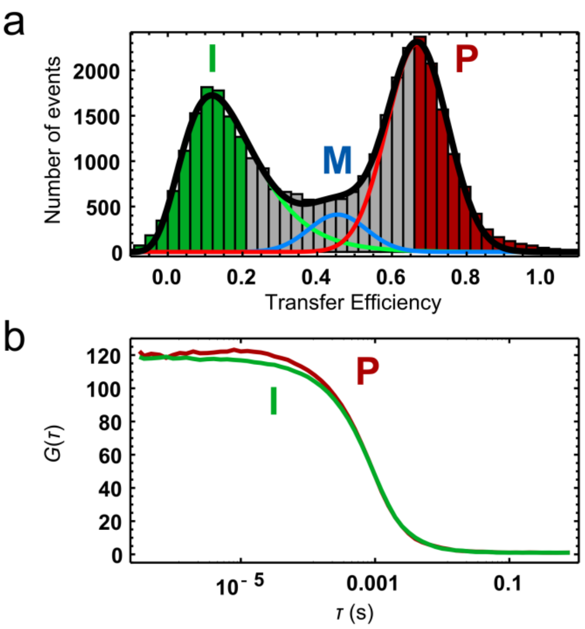

Supplementary Fig. 6. Subpopulation-specific correlation analysis of the ClyA intermediate and protomer. (**a**) Transfer efficiency histogram of labelled ClyAΔCys (0.1 nM, in 0.1 % DDM), in which monomer (M), intermediate (I), and protomer (P) are populated at equilibrium. Solid lines represent the peak fits for the three species present. Fluorescence bursts from the transfer efficiency bins shaded in green and red were used for the subpopulation-specific FCS[1] of the intermediate and protomer state, respectively. (**b**) Correlation curves calculated from the subpopulation-specific acceptor and donor fluorescence signal. The diffusion coefficients (and thus the Stokes radii) of the intermediate and the protomer are equal to within experimental uncertainty. The Stokes radius of the protomer was determined by 2f-FCS to be 4.9±0.1 nm (see main text and Fig. 6).

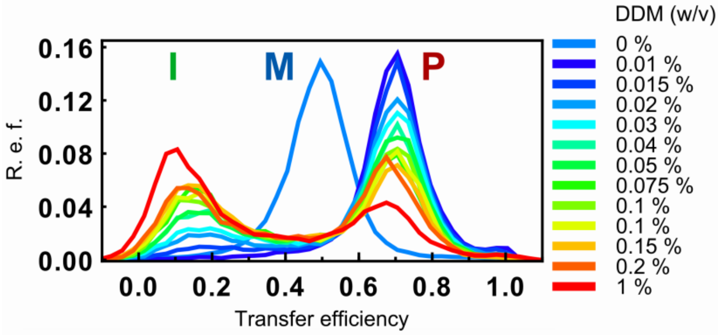

Supplementary Fig. 7. DDM stabilizes the intermediate. The variant ClyAΔCys (0.1 nM), for which the intermediate is stable at equilibrium, was measured in the presence of different concentrations of DDM (as indicated on the right). Without DDM or below the CMC (ca. o.oo8%), only the monomer (M) is present. Increasing amounts of DDM first lead to the population of the protomer (P) and then shifted the equilibrium towards the intermediate (I), concomittant with a slight shift of I to lower transfer efficencies, corresponding to slight expansion of the conformational ensemble. Above 0.15 % DDM, P also shifts to lower transfer efficiency. R. e. f.: relative event frequency.

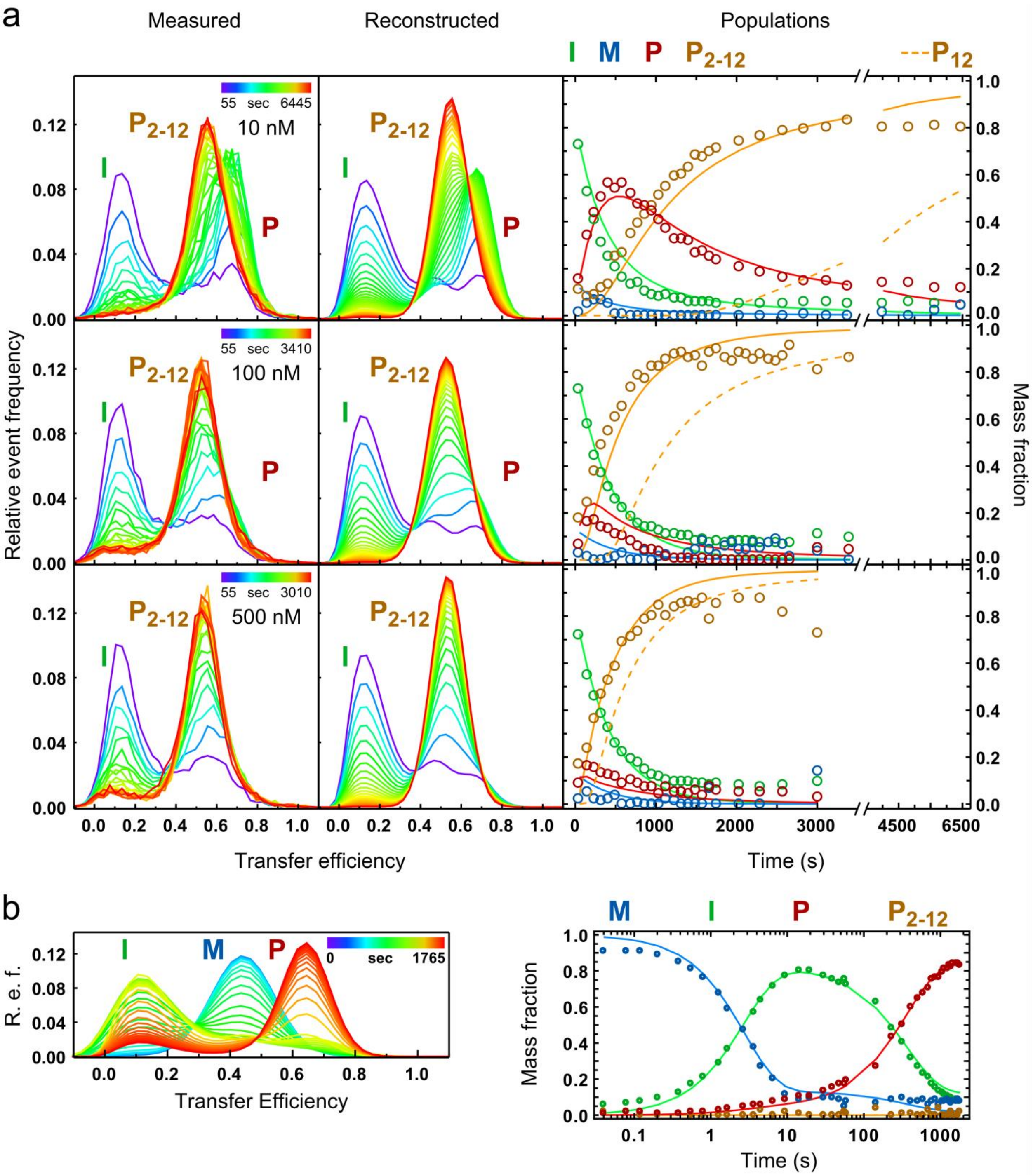

Supplementary Fig. 8. Data of the global fit based on the non-sequential assembly model not shown in Fig. 4. (**a**) Data for the three ClyA concentrations not shown in Fig. 4a-c. Left column: Measured histogram series. Each line represents one transfer efficiency histogram (normalized to an area of 1) at a certain time after mixing the protein with DDM as indicated by the colour (colour code shown in the upper right of each panel). Middle column: Reconstructed histogram series according to the pore formation model shown in Fig. 4d. Right column: Population time courses of the different species

according to two different types of analysis of the histogram time series. Circles: Populations from individual fits of the histograms with peak amplitudes as free parameters and peak positions and widths of all conformational states used as shared (global) fit parameters. Solid lines: Global fit of all 217 histograms according to the non-sequential assembly model with two rate coefficients (see Fig. 4d). The dashed orange lines show the population of complete pores as predicted by the model. The time courses of the individual oligomeric species are shown in Supplementary Fig. 9c. I: intermediate, $P_{2\text{-}12}$: oligomeric species, P: protomer, M: monomer. (**b**) The histogram time series at 100 pM ClyA were also included in the global fit of the assembly kinetics. Left panel: Reconstructed histograms according to the fitted pore formation model. Each line represents one histogram and is colour-coded according to the scale on the upper right. R. e. f.: relative event frequency. Right panel: Population time courses of the different species according to the two fits described in (a). As the different oligomeric species cannot be distinguished in the single molecule data, the plotted species $P_{2\text{-}12}$ represents the sum of all these species. See Methods for details on the fitting procedure. M: monomer, I: intermediate, P: Protomer, $P_{2\text{-}12}$: oligomeric species

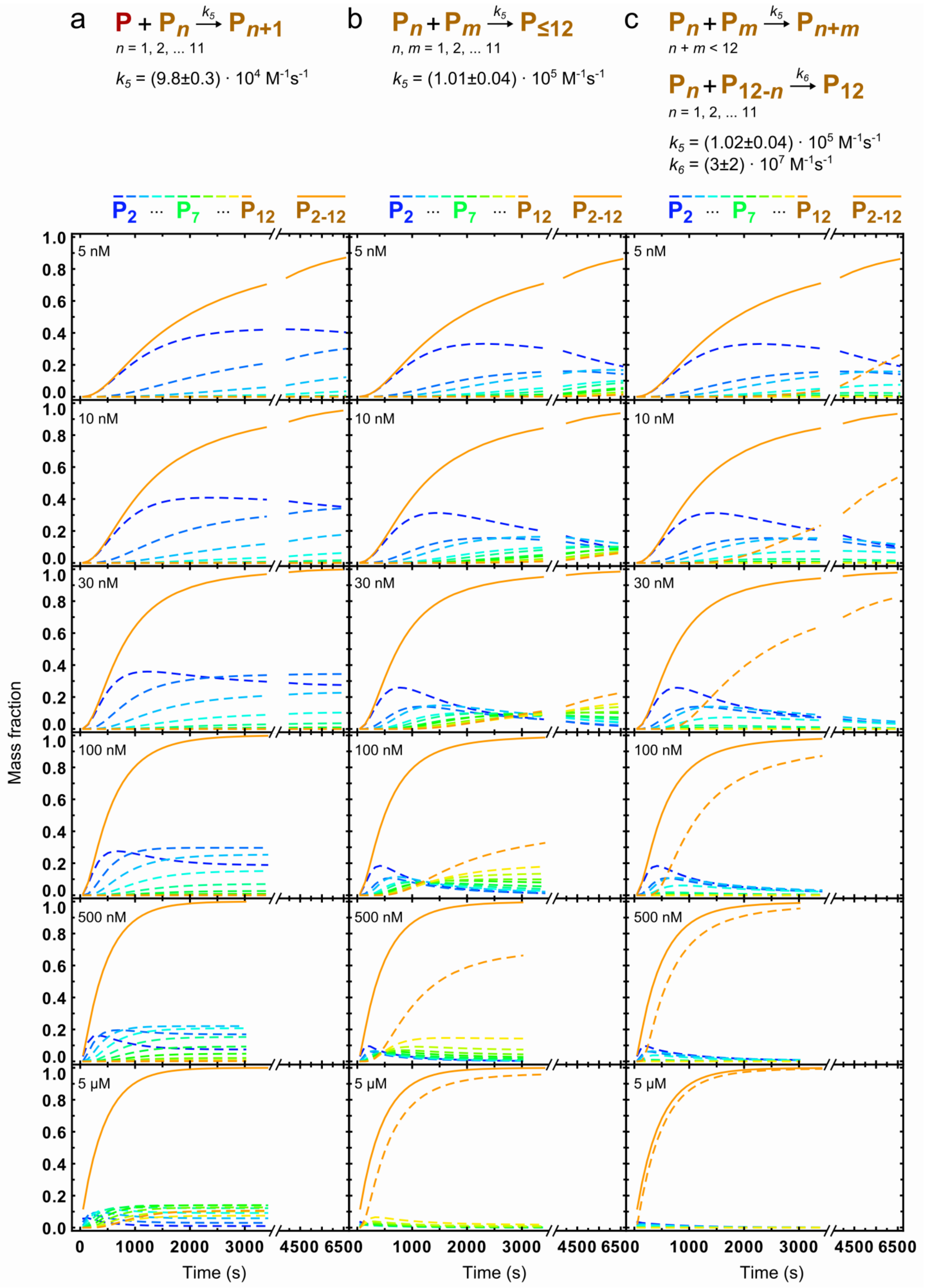

a
$P + P_n \xrightarrow{k_5} P_{n+1}$
n = 1, 2, ... 11
k_5 = (9.8±0.3) · 10^4 $M^{-1}s^{-1}$
b
$P_n + P_m \xrightarrow{k_5} P_{\leq 12}$
n, m = 1, 2, ... 11
k_5 = (1.01±0.04) · 10^5 $M^{-1}s^{-1}$
c
$P_n + P_m \xrightarrow{k_5} P_{n+m}$
n + m < 12
$P_n + P_{12-n} \xrightarrow{k_6} P_{12}$
n = 1, 2, ... 11
k_5 = (1.02±0.04) · 10^5 $M^{-1}s^{-1}$
k_6 = (3±2) · 10^7 $M^{-1}s^{-1}$
P_2 ... P_7 ... P_{12} P_{2-12}
5 nM
10 nM
30 nM
100 nM
500 nM
5 μM
Mass fraction
Time (s)
0 1000 2000 3000 4500 6500

Supplementary Fig. 9. ClyA oligomer and pore populations as predicted by three different kinetic models. (**a**) Linear addition model with a single association rate coefficient (k_5). (**b**) Non-sequential addition model with a single association rate coefficient (k_5). (**c**) Non-sequential addition model with two rate coefficients (k_5, k_6). On top of each column, the oligomerization reaction is depicted schematically. Below, the population time courses of the individual oligomer species (dashed lines, colour code on top) and the total oligomer concentration (solid line) is shown for six different total ClyA concentrations. Note that the linear addition model (a) predicts only a very small amount of complete pores formed even at 5 µM after one hour, and is thus not compatible with our crosslinking and 2f-FCS experiments (Figs. 5, 6). The non-sequential model with one rate coefficient (b) predicts more but still insufficient complete pores. Only with the addition of a second, faster, rate coefficient for the final assembly step (c) does the model yield a sufficiently rapid accumulation of pores to be compatible with the experimental data.

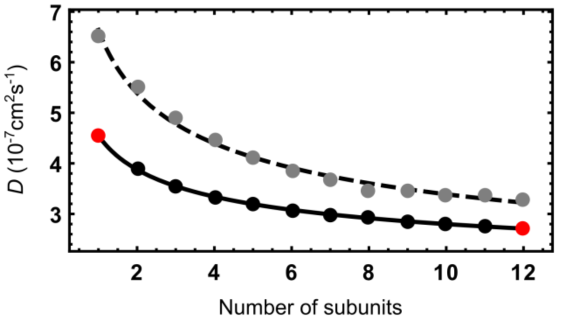

Supplementary Fig. 10. Estimation of the diffusion coefficients of oligomeric ClyA species with different numbers of subunits. Diffusion coefficients (*D*) calculated using HydroPro[2] for the protomer, the intermediary oligomers (P_2 to P_{11}), and the pore (grey dots) can be described by an $n^{-1/3}$ scaling (dashed line), with *n* being the number of subunits in the oligomer (see Methods, 2f-FCS fitting procedure and modelling). The discrepancy between calculated and measured values (red dots) is most likely caused by DDM bound to the protein, and we interpolate between the two values also with an $n^{-1/3}$ scaling to obtain the diffusion coefficients of the intermediate oligomers (black dots).

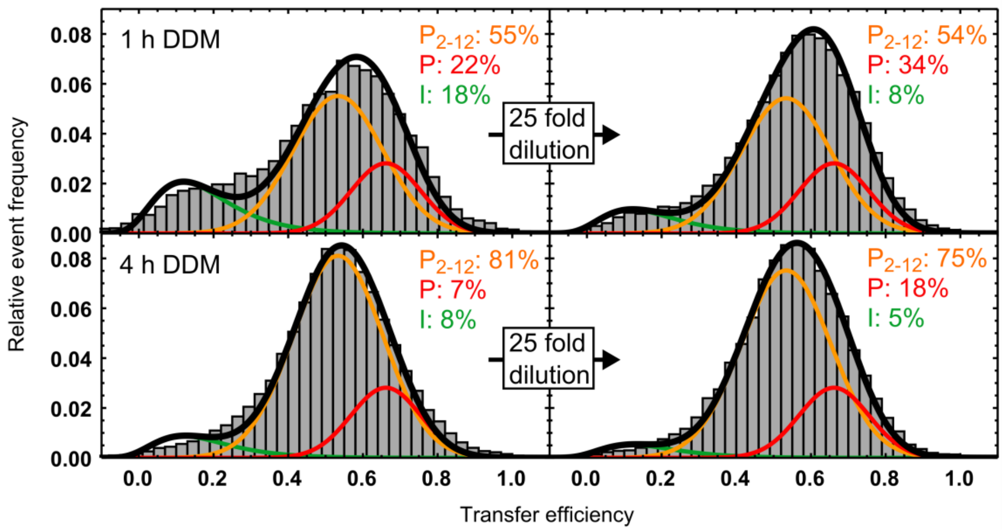

Supplementary Fig. 11. Dissociation of ClyA oligomers is negligible during the experimental observation time. ClyA oligomers were formed from 5 nM ClyAwt and 0.5 nM labelled ClyA in 0.1 % (w/v) DDM for 1 h (top left: last 5 min) and 4 h (bottom left: last 30 min) before diluting the samples 25 x into the same buffer and measuring for 14 to 16 h (right). Histograms were fit with peak positions and widths determined from reference measurements of the different species. Percentages of oligomers ($P_{2\text{-}12}$) and protomer (P) are given as the percentage of bursts in the fitted peak relative to the total number of bursts in the histogram. The results indicate that dissociation of oligomers on the timescales investigated in the assembly kinetics is negligible.

	ClyA A488		ClyA A488/A594				Transfer efficiency based on		
	A488		A488		A594		FRET efficiency histogram	Donor fluores-cence lifetime	Crystal structures (Cα – Cα distances)
	τ	r	τ	r	τ	r			
Protomer	4.0	0.16	1.0	0.18	4.0	0.18	0.72±0.03	0.75±0.03	0.74 (45.5 Å)
Pore	4.0 (4.0)	0.18 (0.18)	1.2	0.21	4.0 (4.2)	0.27 (0.29)	0.57±0.03	0.70±0.03	0.74 (45.5 Å)

Supplementary Table 1: Acceptor dye quenching is most likely responsible for the difference in transfer efficiency between the protomer and the pore. τ: Fluorescence lifetimes, r: polarisation anisotropies, A488: Alexa Fluor 488, A594: Alexa Fluor 594. Transfer efficiencies are determined in the following way. FRET efficiency histograms: average of the Gaussian peak function fits to single-molecule FRET transfer efficiency histogram (see Methods section). Donor fluorescence lifetime: calculated from the difference in donor fluorescence lifetime in the absence ($\tau_{ClyAA488}$) and presence ($\tau_{ClyAA488/594}$) of acceptor ($E = 1- \tau_{ClyAA488/594}/\tau_{ClyAA488}$). Crystal structures: calculated using the Cα – Cα distances of the labelling positions in the crystal structures (PDB codes 1QOY and 2WCD) and the Förster equation with $R_0 = 5.4\ nm^3$. Note that the transfer efficiency of the protomer is close to the value expected based on the distance in the crystal structure of the pore, indicating that the protomer structure closely resembles the structure of a subunit in the pore. For the pore state, a difference in transfer efficiency compared to the value of $E = 0.74$ expected from the crystal structure is observed in the FRET efficiency histogram. However, this discrepancy can largely be attributed to static quenching of the acceptor dye, because the transfer efficiency obtained from the donor fluorescence lifetime is 0.70, close to the expected value. Note that this difference in apparent transfer efficiency due to acceptor quenching is an essential (albeit fortuitous) prerequisite for separating monomeric protomer and pore conformations of ClyA in the FRET efficiency histograms. All values in the table were determined by single-molecule measurements, except those in parentheses (ensemble measurements). Note that the protomer is not amenable to ensemble methods because it can only be stably populated at sub-nanomolar ClyA concentrations. For details on the measurements, see Methods. For the uncertainties of the transfer efficiencies from histogram data, see Methods; for the fluorescence lifetimes, our estimate of the experimental uncertainly is 0.1 ns, resulting in the standard deviations of the transfer efficiencies indicated in the table.

Total ClyA concentration	Total measurement time	Repeats used	Time window length	Burst min/max
100 pM	60 sec (microfluidics)	NA	NA	35/300
100 pM	30 min	21	90 s	80/500
5 nM	30 min	17	90 s	80/500
5 nM	60 min	7	270 s	80/500
5 nM	120 min	4	540 s	80/500
10 nM	30 min	17	90 s	80/500
10 nM	60 min	6	270 s	80/500
10 nM	120 min	2	540 s	80/500
30 nM	30 min	16	90 s	80/500
30 nM	60 min	8	270 s	80/500
30 nM	120 min	2	540 s	80/500
100 nM	45 min	18	90 s	80/500
100 nM	60 min	4	400 s	80/500
500 nM	30 min	18	90 s	80/500
500 nM	45 min	6	270 s	80/500
500 nM	60 min	2	400 s	80/500
5 µM	40 min	12	90 s	80/500
5 µM	60 min	3	400 s	80/500

Supplementary Table 2: Data sets collected for the kinetic analysis for ClyA protomer and pore formation. Repeats used: number of measurements taken for the specified time; Time window length: size of time windows that the cumulated measurements were divided into; Burst min/max: lower and upper limit of number of photons in a burst to be considered for the analysis; NA: not applicable.

Supplementary References

1. Laurence TA, Kwon Y, Yin E, Hollars CW, Camarero JA, Barsky D. Correlation spectroscopy of minor fluorescent species: Signal purification and distribution analysis. *Biophysical Journal* **92**, 2184-2198 (2007).

2. Ortega A, Amoros D, de la Torre JG. Prediction of Hydrodynamic and Other Solution Properties of Rigid Proteins from Atomic- and Residue-Level Models. *Biophysical Journal* **101**, 892-898 (2011).

3. Schuler B, Lipman EA, Eaton WA. Probing the free-energy surface for protein folding with single-molecule fluorescence spectroscopy. *Nature* **419**, 743-747 (2002).
